# SIRF: Quantitative in situ analysis of protein interactions at DNA replication forks

**DOI:** 10.1083/jcb.201709121

**Published:** 2018-04-02

**Authors:** Sunetra Roy, Jessica W. Luzwick, Katharina Schlacher

**Affiliations:** Department of Cancer Biology, University of Texas MD Anderson Cancer Center, Houston, TX

## Abstract

DNA replication reactions are central to diverse cellular processes including development, cancer etiology, drug treatment, and resistance. Many proteins and pathways exist to ensure DNA replication fidelity and protection of stalled or damaged replication forks. Consistently, mutations in proteins involved in DNA replication are implicated in diverse diseases that include defects during embryonic development and immunity, accelerated aging, increased inflammation, blood disease, and cancer. Thus, tools for efficient quantitative analysis of protein interactions at active and stalled replication forks are key for advanced and accurate biological understanding. Here we describe a sensitive single-cell–level assay system for the quantitative analysis of protein interactions with nascent DNA. Specifically, we achieve robust in situ analysis of protein interactions at DNA replication forks (SIRF) using proximity ligation coupled with 5′-ethylene-2′-deoxyuridine click chemistry suitable for multiparameter analysis in heterogeneous cell populations. We provide validation data for sensitivity, accuracy, proximity, and quantitation. Using SIRF, we obtained new insight on the regulation of pathway choice by 53BP1 at transiently stalled replication forks.

## Abstract (precis)

Roy et al. describe a novel assay to measure direct protein associations at active and stalled DNA replication forks, called in situ analysis of protein interactions at DNA replication forks. The EdU-chase, click-chemistry, and PLA-composite system is quantitative, sensitive, and effective, with single-cell resolution suitable for concomitant multiparameter analysis.

## Introduction

DNA replication and its regulations dictate outcomes of many biological processes including development, aging, and cancer etiology (Loeb and Monnat, 2008; Zeman and Cimprich, 2014). DNA is continuously subject to damage challenging the maintenance of the genome code and stability. Consistently, genome instability is associated with cancer etiology, and DNA replication errors are the most frequently found cause for cancer mutations (Hanahan and Weinberg, 2011; Tomasetti et al., 2017). Thus, cells contain intricate protection pathways for replication reactions to ensure faithful and complete replication of the genome.

DNA protection pathways engage proteins acting directly during DNA replication, including replisome components such as DNA polymerases (Loeb and Monnat, 2008). Yet a rapidly evolving and exciting field is the direct involvement of proteins during DNA replication that are otherwise understood to repair DNA damage irrespective of DNA replication. Among others, these include BRCA1/2 and Fanconi anemia tumor suppressors, which protect stalled DNA replication forks from degradation by MRE11 and DNA2 nucleases and so suppress genome instability (Schlacher et al., 2011, 2012; Pefani et al., 2014; Higgs et al., 2015; Wang et al., 2015; Ding et al., 2016; Ray Chaudhuri et al., 2016). Although a body of evidence clearly delineates the importance of DNA repair proteins for mending DNA breaks after physical DNA damage (Moynahan and Jasin, 2010; Roy et al., 2011; Ceccaldi et al., 2016), this ever-growing list of classic DNA repair proteins acts directly in protecting DNA replication forks from damage.

Cellular signaling pathways also have a direct impact on DNA replication. This includes, most prominently, cell cycle control pathways (Petermann et al., 2010b; Guo et al., 2015; Galanos et al., 2016). Recent publications link signaling pathways with functions in the cytoplasm to the regulation of DNA replication reactions. This involves a YAP-1 independent function of the Hippo pathway in protecting nascent DNA forks from degradation by MRE11 and so promoting genome stability (Pefani et al., 2014). Another example is the phosphatase and tensin homolog ten, PTEN, which is the second most frequently mutated tumor suppressor and best understood for its phosphatase activity in regulating the cytoplasm membrane-bound phosphoinositide 3-kinase kinase pathway (Stiles et al., 2004; Song et al., 2012). Yet PTEN has a nuclear function in promoting genome stability and regulating DNA replication restart reactions (He et al., 2015). Moreover, DNA replication reactions are the targets of most standard-of-care chemotherapy strategies and as such intricately involved with mechanisms for acquiring drug resistance (Ding et al., 2016; Ray Chaudhuri et al., 2016). Thus, efficient and effective molecular tools allowing fine-scale resolution and quantitation of DNA replication reactions and protein interactions at nascent DNA replication forks are critical for advances in the molecular and cellular understanding of nontraditional DNA replication proteins and pathways.

The development of single-molecule resolution assays for studying DNA replication and repair is enabling the advancement of our understanding of replication reactions. Examples include single-molecule DNA spreading and genome combing techniques allowing the quantitative assessment of genome-wide replication speeds and perturbations (Michalet et al., 1997; Jackson and Pombo, 1998; Técher et al., 2013). Another notable ground-breaking technology was the development of isolation of proteins on nascent DNA (iPOND), which allows for high-resolution analysis of proteins at replication forks (Petermann et al., 2010a; Sirbu et al., 2011, 2012). In brief, nascent DNA is labeled by incorporation of a thymidine analogue such as 5′-ethylene-2′-deoxyuridine (EdU) during tissue cell culture. After cell fixation, EdU is conjugated with biotin using click chemistry. Genomic DNA then is isolated and sheared by sonication, and nascent DNA fragments of ∼100–300 base pairs are pulled down using streptavidin beads. Proteins cross-linked to the biotinylated DNA fragments then can be resolved by Western blot analysis (Sirbu et al., 2011, 2012). A valuable extension of this technology uses stable isotope laleling with amino acids in cell culture (SILAC; Sirbu et al., 2013; Cortez, 2017), where the candidate approach by Western blot analysis is replaced with a discovery-based approach by mass-spectrometry analysis, allowing for refined, sensitive, and unbiased protein detection. These technologies have revolutionized our understanding of DNA replication reactions and unveiled many reactions that so far were mysterious because of lack of the molecular resolution. These fine-resolution methods are valuable, but they are also laborious, requiring advanced and specialized technical skills and machinery, which considerably limits efficient progress. Moreover, iPOND requires liberal amounts of starting material (∼100,000,000 cells per condition), measures cell population means that are blind to heterogeneous cell changes, has limited sensitivity associated with challenging quantitation (Western blot), or, in the case of SILAC, requires high-cost specialized equipment with limited access (mass-spectrometry analysis). We here describe an assay system termed in situ protein interactions at nascent and stalled replication forks (SIRF) that uses proximity ligation assay (PLA) technology and overcomes these challenges; SIRF allows for efficient analysis of protein interactions at nascent replication forks on a single-cell level. It is readily quantifiable, requires very little starting cell material, is sensitive, and can be accomplished with equipment found standard in molecular biology laboratories. Importantly, SIRF has single-cell resolution that can provide added information including cell identity and spatial localization, allowing studies of heterogeneous cell populations, and so valuably can be used as a multiparameter assay. Thus, SIRF is an enabling technology for fine-scale understanding of DNA replication processes in single cells and diverse cell populations.

## Results

### SIRF procedure

**Figure 1.**
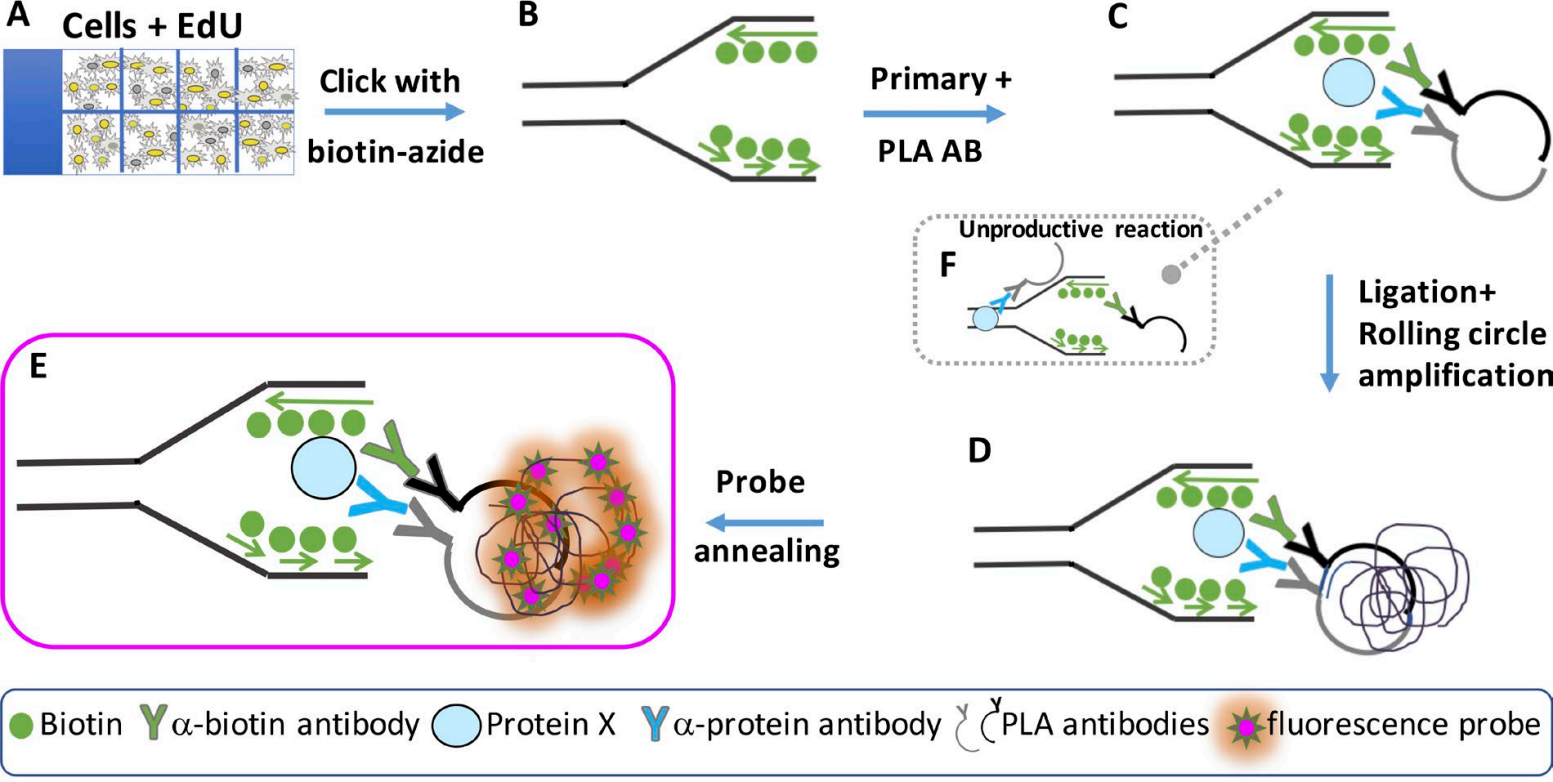
**Schematic representation outlining SIRF assay. (A)** Cells are grown in microscope chamber-slides and pulsed with EdU. **(B)** EdU is biotinylated using click chemistry. **(C)** Slides are incubated with primary antibody (AB) against protein of interest and against biotin, followed by incubation with secondary PLA antibodies containing a DNA-oligomers. **(D)** A linker DNA binds to the antibody-oligomers allowing T7-mediated ligation for rolling circle amplification by PHI29 polymerase. **(E)** A fluorescent DNA probe anneals in a sequence specific fashion to the amplification product, thus producing many red fluorescent signals per one antibody interaction, which results in robust and detectable fluorescence. **(F)** Unproductive reaction occurs when the PLA antibodies are not in close proximity, thus inhibiting formation of circular template and consequent fluorescent probe annealing of the amplified circle.

SIRF technology allows for the quantitative assessment of protein interactions with ongoing, stalled, and previously active replication forks using PLA technology (Fig. 1). Specifically, ∼10,000 cells are grown on coverslips or in microscope-slide chambers and pulse-labeled with the nucleotide analogue EdU for 8 min (Fig. 1 A). EdU is an alkyne group containing thymidine analogue that is incorporated into newly synthesized DNA and so marks sites of nascent DNA. EdU can be chased with thymidine to study active and previously active replication fork associations or with replication stalling agents including hydroxyurea (HU) to study protein associations with stalled replication forks. Proteins in proximity are then covalently cross-linked to nascent DNA by incubation with paraformaldehyde. After cell permeabilization, click chemistry between the alkyne group of EdU and a biotin-azide allows for the biotinylation of the EdU-incorporated DNA (Fig. 1 B). Cells are subsequently incubated with primary antibodies against biotin and the protein of interest (Fig. 1 C). The protocol then follows the principle of the PLA (Söderberg et al., 2006), and cells are incubated with secondary antibodies conjugated to sequence specific DNA oligomers (Fig. 1 C, PLA antibodies). If the PLA antibodies are in proximity of less than 40 nm, typifying direct interaction, the DNA oligomers are able to anneal with a linker oligomer, forming a nicked circular DNA. Ligation of the nick with T7 ligase enables rolling circle replication by Phi29 polymerase, which amplifies the DNA sequence ∼100-fold (Fig. 1 D). DNA sequence–specific fluorescence DNA probes then anneal to the amplified DNA circles. Through rolling circle DNA amplification, a single epitope–epitope interaction is marked with ∼100 fluorescent probes. This creates a strong fluorescence signal and increases sensitivity to enable detection of single protein interaction, and in the case of SIRF, protein-nascent DNA interactions (Fig. 1 E). The signal is specific as it relies on tight proximity of the secondary antibody DNA conjugates. As an extension in the SIRF procedure, no signal is obtained if the protein of interest is not in close proximity to the nascently labeled DNA (Fig. 1 F).

### EdU distance and SIRF signal

**Figure 2.**
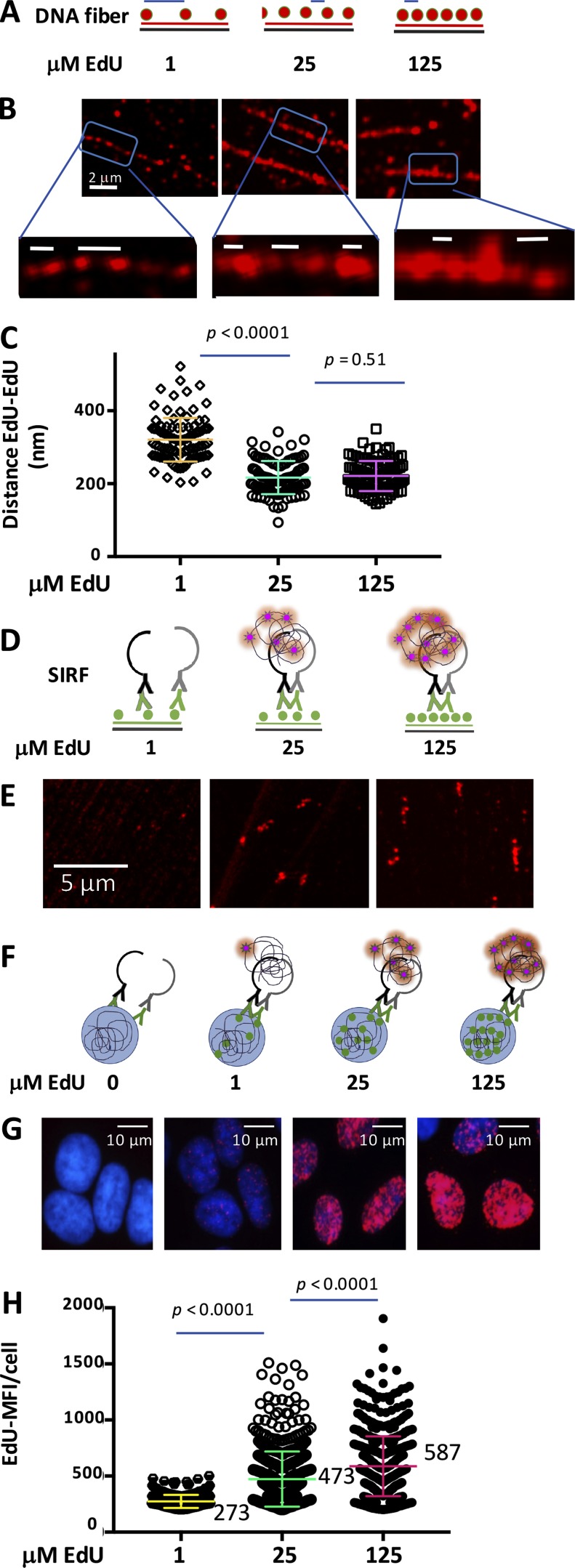
**EdU incorporation frequency necessary for productive SIRF. (A)** Graphical sketch of EdU-incorporated DNA fibers. The distances between EdU nucleotides varies with EdU concentration and is measured (blue line). **(B)** Representative Airyscan images of single-molecule DNA fibers at 1, 25, and 125 µM EdU in HAP-1 cells using anti-biotin antibodies against biotinylated EdU. **(C)** Scatter plot of distances between EdU signals in DNA fibers obtained using Airyscan superresolution microscopy in HAP-1 cells at varying EdU concentrations as indicated. **(D)** Graphical sketch of productive PLA-EdU-DNA fiber signal dependence on EdU concentrations. **(E)** Representative Airyscan images of single-molecule DNA fibers at 1, 25, and 125 µM EdU in HAP-1 cells using PLA against biotinylated EdU. **(F)** Graphical sketch of productive PLA-SIRF signal dependence on EdU concentration. **(G)** Representative images of HAP-1 cells treated with 0, 1, 25, and 125 µM EdU. **(H)** Scatter plot of EdU-SIRF signals in HAP-1 cells with 1, 25, and 125 µM EdU. Bars represent the mean and SD of combined data from repeated experiments. The significance values are derived from Mann-Whitney statistical analysis.

A successful signal production by PLA technology used in the SIRF assay is based on a maximal proximity of ∼40 nm between two epitopes (Söderberg et al., 2006). We first sought to determine the relationship between DNA epitope proximity and SIRF signals. We measured the distance between nascent incorporated EdU nucleotides (Fig. 2 A). HAP-1 cells were exposed to varying concentrations of EdU, DNA fiber spreading was performed to visualize single DNA replication tracts and, after a click reaction, biotinylated EdU was detected with Neutravidin-Texas red (Fig. 2 B). The distance between the single EdU-biotin fluorescence signals was measured by Airyscan superresolution microscopy (Sheppard et al., 2013; Sivaguru et al., 2016; Fig. 2 B). At 1 µM EdU, the distances between EdU signals measured between 203 and 521 nm (Fig. 2 C). At 25 and 125 µM EdU, the distances between EdU signals measured between 94 and 342 nm and 144 and 350 nm, respectively (Fig. 2 C), thus reaching the maximal resolution of Airyscan superresolution microscopy (∼120 nm).

We next sought to determine the optimal EdU concentration necessary to obtain a robust SIRF-PLA signal. We performed PLA on DNA fibers against EdU-biotin using mouse–α-biotin and a rabbit–α-biotin antibody (Fig. 2 D). No PLA fiber signals were detected at 1 µM EdU (Fig. 2 E). This is consistent with our DNA fiber data by immunofluorescence (IF) showing EdU epitope spacing >200 nm (Fig. 2 C), which is too far apart for a productive PLA signal. At 25 µM EdU, PLA signals from single fibers were obtained, albeit we observed visibly more robust PLA fiber signals with 125 µM EdU (Fig. 2 E).

In cells, DNA is folded into chromatin, which may affect the relative distance between EdU molecules within the 3D architecture. We therefore performed SIRF assays in human HAP-1 cells against EdU (Fig. 2 F). As a control, omission of EdU did not result in any SIRF-PLA signals (Fig. 2 G). We quantified the EdU-SIRF signals by measuring the mean fluorescence intensity (MFI) per cell as the signals were too abundant and fused, and therefore could not be counted individually. At 1 µM EdU, there were very weak SIRF-PLA signals (Fig. 2 H, mean MFI/cell of 273). At 25 µM EdU, robust PLA signals were obtained (Fig. 2 H, mean MFI/cell of 473). Nevertheless, we observed significantly increased SIRF-PLA signals with 125 µM EdU (Fig. 2 H, mean MFI/cell of 587, P *<* 0.0001). These results suggest that increased EdU concentrations result in more frequently incorporated EdU, thus less distance between each EdU moiety and increased SIRF-PLA signals. From the collective results, we conclude that higher EdU concentrations achieve closer proximity with the target epitope and thus higher sensitivity in the SIRF assay.

### SIRF for quantitative detection of active replisomes

**Figure 3.**
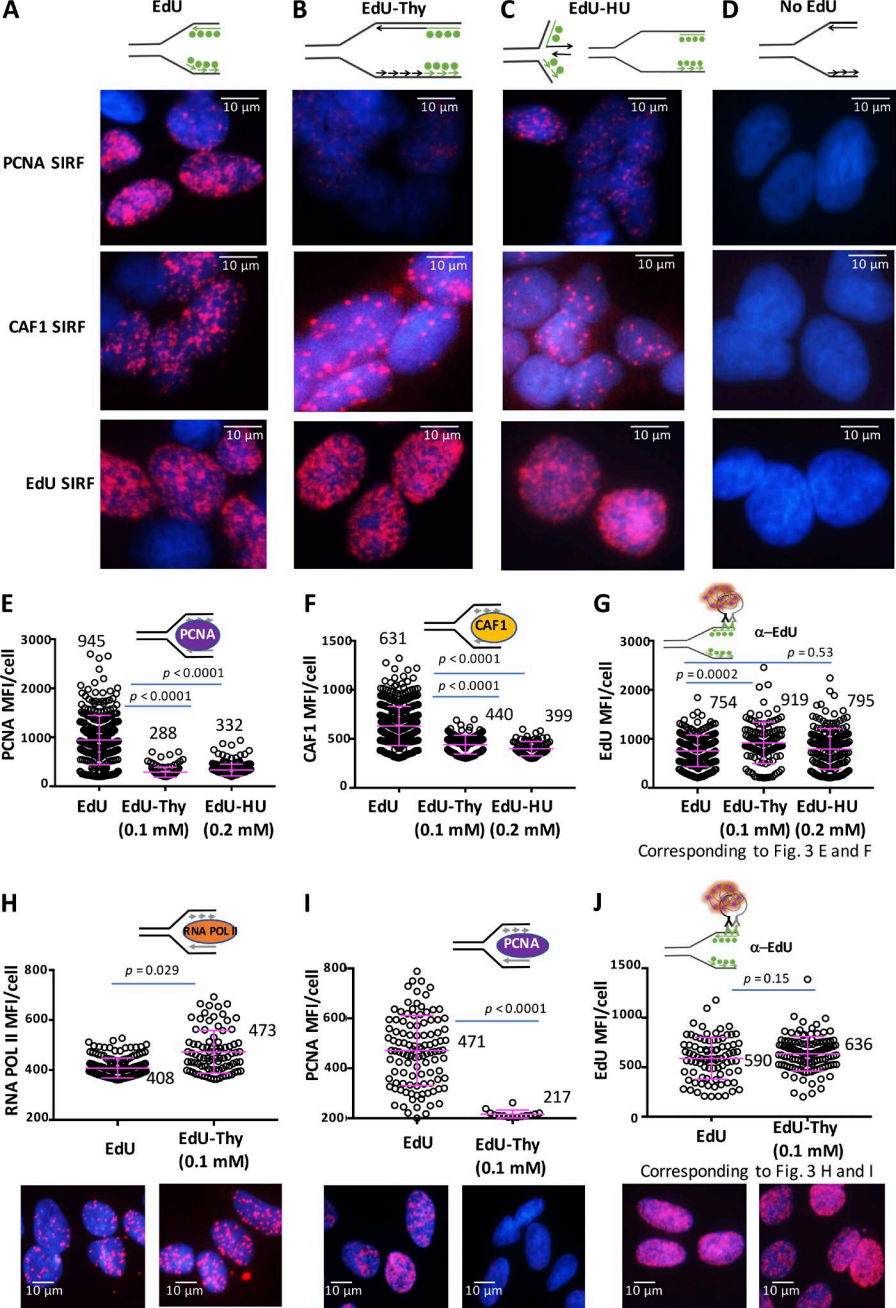
**SIRF detection of replisome components at active replication forks. (A–D)** Representative images of PCNA-, CAF1- and EdU-SIRF in HAP-1 cells treated with EdU (A), EdU followed by thymidine (Thy, 0.1 mM; B), or EdU followed by HU (0.2 mM) for 4 h (C), or not treated (D). **(E)** Scatter plot of PCNA-SIRF signals in unperturbed HAP-1 cells and cells treated with EdU followed by thymidine or HU for 4 h, as indicated. **(F)** Scatter plot of CAF1-SIRF signals in unperturbed HAP-1 cells and cells treated with EdU followed by thymidine or HU, as indicated. **(G)** Scatter plot of EdU-SIRF signals in unperturbed HAP-1 cells and cells treated with EdU followed by thymidine or HU, as indicated. **(H)** Scatter plot of RNA POL II–SIRF signals in unperturbed HAP-1 cells and cells treated with EdU followed by 0.1 mM thymidine (3 h). **(I)** Scatter plot of PCNA-SIRF signals in unperturbed HAP-1 cells and cells treated with EdU followed by 0.1 mM thymidine (3 h). **(J)** Scatter plot of EdU-SIRF signals in unperturbed HAP-1 cells and cells treated with EdU followed by 0.1 mM thymidine (3 h). Bars represent the mean and SD of combined data from repeated experiments. The significance for EdU-SIRF values is derived from the Mann-Whitney statistical test, and the significance for protein-SIRF values is derived from the Mann-Whitney statistical test after normalization to the corresponding EdU-SIRF (see Materials and methods).

We next tested if SIRF can distinguish between proteins at active DNA replication forks and proteins bound to chromatin. To do this we chased the EdU pulse with low concentrations of thymidine before fixing the cells (Fig. 3). Because 125 µM EdU resulted in the most optimal SIRF signal, we used 125 µM EdU from here on unless indicated otherwise. With a thymidine chase, the previously incorporated EdU is no longer present at an active replication fork (Fig. 3 B, top; Sirbu et al., 2011). Thus, replisome components will lose proximity with the biotinylated EdU. Consistently, we observe a stark decrease in SIRF signals against the DNA polymerase processivity factor proliferating cell nuclear antigen (PCNA) and histone remodeler CAF-1 after thymidine chase (Fig. 3, A, B, E, and F; mean PCNA-SIRF MFI/cell of 945 with active forks compared with mean PCNA-SIRF MFI/cell of 288 with thymidine chase, and mean CAF1-SIRF MFI/cell of 631 with active forks compared with mean CAF1-SIRF MFI/cell of 440 with thymidine chase). Similar to the chase with thymidine, replisome components dissemble from transiently stalled or collapsed replication forks (Trenz et al., 2006; Sirbu et al., 2011; Fig. 3, C, E, and F; and Fig. S1 B; mean PCNA-SIRF MFI/cell of 332 and mean CAF1-SIRF MFI/cell of 399 with transiently stalled forks). Importantly, the decreased signal is not a result of decreased EdU incorporation (Fig. 3 G; mean EdU-SIRF MFI/cell of 754 with active forks, mean EdU-SIRF MFI/cell of 919 with thymidine chase, and mean MFI/cell of 795 with 0.2 mM HU). As the concentration of the incorporated EdU can affect the amount of SIRF signal, we normalized the SIRF signals to mean EdU-SIRF signals before statistical analysis (see Materials and methods).

To further validate the SIRF assay for analyzing protein associations at active replication forks, we measured RNA POL II associations to nascent DNA, which is not expected to be associated with active replication forks (Fig. 3 H). In contrast to replisome component PCNA (Fig. 3, I and J), RNA POL II associations with EdU-labeled DNA increase with thymidine chase (Fig. 3 H). These data suggest that RNA POL II does not travel with the replication fork but accumulates after the replisome has moved past the region of DNA, consistent with a previous study that show nascent DNA-RNA hybrid accumulation by PLA proximity ∼30–45 min after DNA replication (Petruk et al., 2016). Collectively, the data validate SIRF for robust objective measurement of protein associations with active replication forks.

### Sensitivity of SIRF

**Figure 4.**
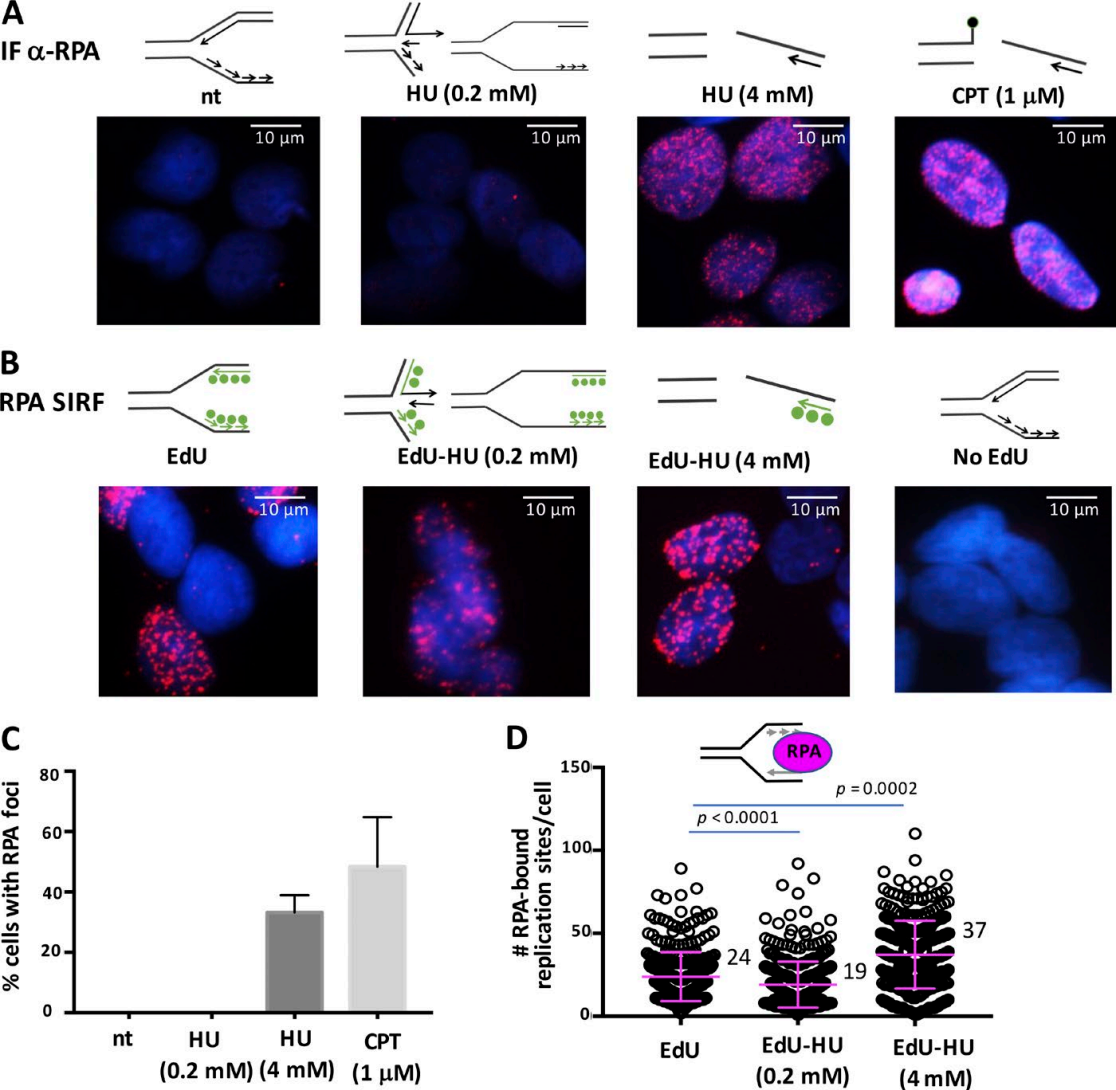
**RPA-SIRF comparison with IF. (A)** Representative images of IF staining for RPA (red) and DAPI (blue) in untreated HAP-1 cells and cells treated with HU or camptothecin (CPT) for 4 h, as indicated. Top: DNA fork structures reported for the corresponding treatments. **(B)** Representative images of RPA-SIRF in HAP-1 cells treated with EdU or EdU followed by HU for 4 h, as indicated. Top: DNA fork structures reported for the corresponding treatments. Green circles represent incorporated EdU. **(C)** Column bar graph of percentage of cells containing IF RPA foci with different treatment conditions as indicated, corresponding to Fig. 3 A. nt, not treataed. Error bars represent SEM of combined data from two biological experiments. A minimum of six image fields was acquired for each condition and for each experiment. **(D)** Scatter plot of RPA-SIRF signals in unperturbed HAP-1 cells and cells treated with EdU followed by HU, as indicated. Bars represent the mean and SD of combined data from repeated experiments. The significance values are derived from Mann-Whitney statistical analysis after normalization to the corresponding EdU-SIRF.

Because of the ease of use, so far the most commonly used technology for assessing the association of DNA replication and repair protein with DNA remains inference by IF imaging of proteins foci or “repair foci” (Scully et al., 1997; Stiff et al., 2004). This technique often uses preextraction of unbound soluble proteins before fixing cells, so that DNA-bound proteins will appear as sharp fluorescent foci after staining with an IF antibody (Fig. 4 A). This technique has proven tremendously informative for the analysis of protein recruitment to damaged and broken DNA (Bekker-Jensen and Mailand, 2010). We therefore used this technique as an added measure to test the sensitivity of SIRF detection of DNA replication and repair proteins at replication forks. We measured the foci frequency of the replication protein A (RPA), which binds single stranded DNA, and found that under conditions that elicit broken DNA (Fig. S1 B; Zellweger et al., 2015), which include treatment with high concentrations of HU or camptothecin, RPA can be prominently detected in damage-induced foci (Fig. 4, A and C). However, the IF method failed to readily detect RPA fluorescence foci by IF with low HU concentrations, which result in transiently stalled replication forks without considerable breakage (Fig. S1 B; Zellweger et al., 2015), or under unchallenged conditions (Fig. 4, A and C). As RPA is an integral protein necessary during Okazaki fragment synthesis, our data suggest that by common IF, it is challenging to robustly detect RPA under physiological unperturbed conditions, where RPA is likely less abundant compared with RPA found at damaged DNA sites.

We therefore tested SIRF against RPA in unchallenged cells and cells exposed to HU, which stalls DNA replication forks (Fig. 4, B and D). Importantly and in sharp contrast to IF, SIRF analysis readily resulted in robust RPA-SIRF signals, signifying RPA bound to unchallenged replication forks (Fig. 4, B and D; and Fig. S1, A and D). Similarly, RPA-SIRF in HAP-1 cells with transiently stalled replication forks resulted in appreciable SIRF signals, which could not be detected by IF, albeit it was reduced compared with unchallenged cells (Fig. 4 D). The reduced RPA-SIRF signal was not a result of reduced EdU incorporation (Fig. S1 D). In contrast to low-dose HU, high concentrations of HU can result in broken DNA replication forks (Fig. S1 B; Zellweger et al., 2015). With high concentrations of HU, we find an appreciable increase in RPA signal compared with low concentrations of HU, consistent with data obtained by IF (Fig. 4, B and D; and Fig. S1). No RPA signals are detected with SIRF analysis when EdU is omitted, confirming the specificity of the signal (Fig. 4 B). Comparing RPA-SIRF signals at high HU and no HU conditions does not result in a dramatic signal increase, as would be expected from IF studies (Fig. 4 A). Using concentrations of 1 and 25 µm EdU resulted in lower RPA-SIRF signals compared with 125 µm EdU (Fig. S1, C and D). Because RPA is binding to the parental DNA strands after degradation of the nascent DNA, and lower EdU concentrations decrease the incorporation frequency and so the proximity to the end of the nascently labeled DNA (Fig. 2 B), we conclude that the SIRF-RPA signals represent interactions with the tip of the nascent DNA strand. This results in a relatively smaller increase in signals, as is expected from the IF data, which measures total RPA association, including with parental DNA strands. Collectively, the data suggest that, in contrast to IF, SIRF is able to detect protein interactions with ongoing and transiently stalled replication fork ends.

**Figure 5.**
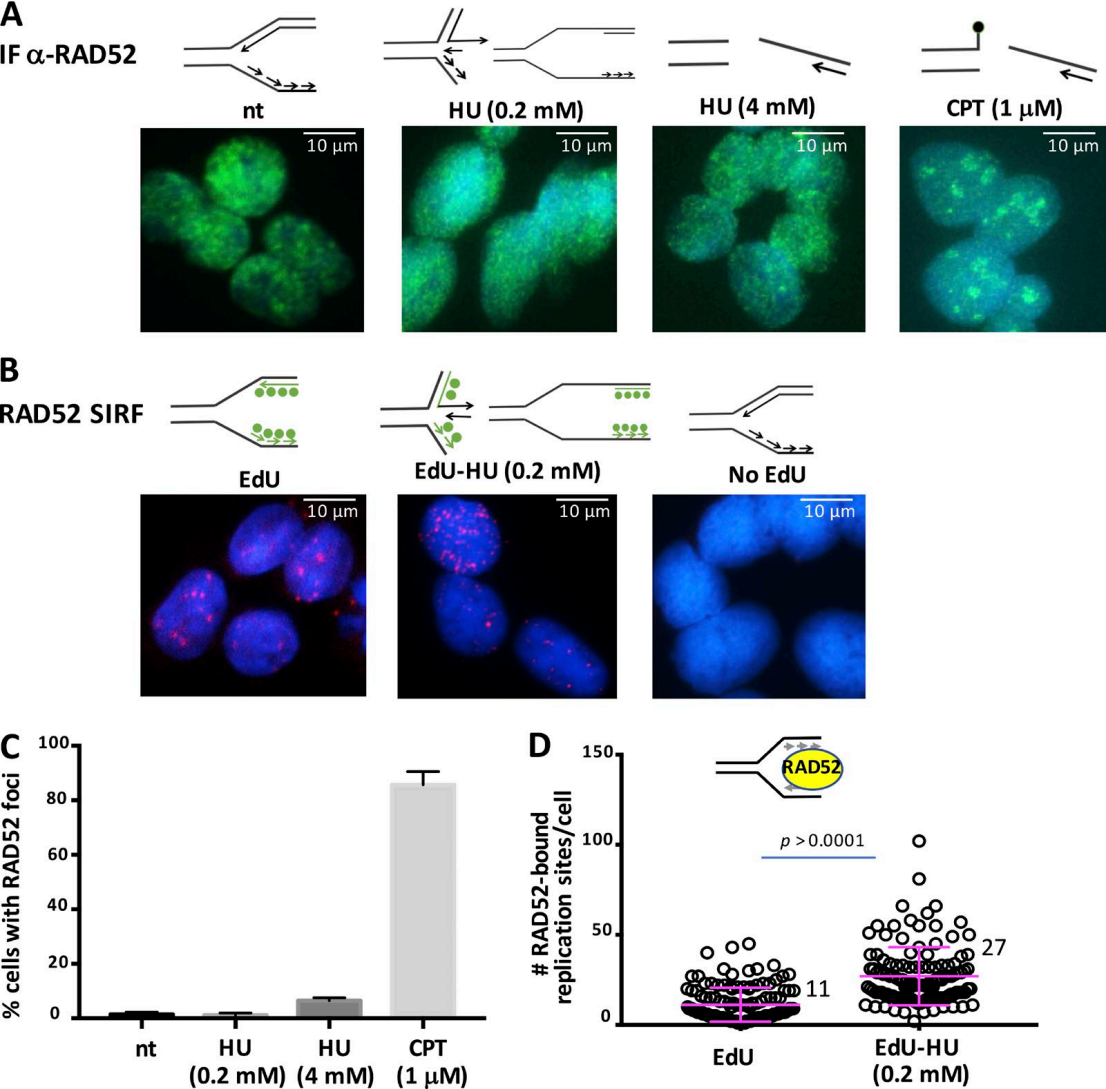
**RAD52-SIRF comparison with IF. (A)** Representative images of IF staining for RAD52 (green) and DAPI (blue) in untreated HAP-1 cells and cells treated with HU or camptothecin (CPT) for 4 h, as indicated. Sketches on top represent DNA fork structures reported for the corresponding treatments. See also Fig. S1 B for DNA structures. **(B)** Representative images of RAD52-SIRF in HAP-1 cells treated with EdU or EdU followed by HU, as indicated. Top: DNA fork structures reported for the corresponding treatments. Green circles represent incorporated EdU. **(C)** Column bar graph of percentage of cells containing IF RAD52 foci with different treatment conditions as indicated, corresponding to Fig. 3 A. Error bars represent SEM of combined data from two biological experiments. A minimum of six image fields was acquired for each condition and for each experiment. **(D)** Scatter plot of RAD52-SIRF signals in unperturbed HAP-1 cells and cells treated with EdU followed by HU, as indicated. Bars represent the mean and SD of combined data from repeated experiments. The significance values are derived from Mann-Whitney statistical analysis after normalization to the corresponding EdU-SIRF.

DNA repair proteins are increasingly found to interact, protect, and promote DNA replication fork reactions. We therefore further sought to test if SIRF is capable of detecting DNA repair proteins unseen by IF. We first determined the frequency of repair foci of RAD52, a protein involved in single-strand annealing (SSA), which is a mutagenic double-strand break (DSB) repair pathway (Fig. 5 A). Similar to RPA, we detect repair foci with camptothecin and high concentrations of HU, but not at unchallenged or transiently stalled replication forks (Fig. 5, A and C). Using RAD52 SIRF, we find RAD52 associates with unchallenged replication forks, and this association is increased with transient replication stalling by low HU concentrations (Fig. 5, B and D; and Fig. S2 B). As with RPA, we confirmed that lower EdU concentrations did not increase but rather decreased the sensitivity of the RAD52-SIRF assay (Fig. S2 A). To further test these data, we independently performed RAD52-SIRF in MDA-MB-231 breast cancer cells and in MCF10A, which are spontaneously immortalized normal mammary epithelial cells (Fig. S2, C–F). As with HAP-1 cells, we readily find the repair factor RAD52 recruited to transiently stalled replication forks (Fig. S2, A, C, and E). The data suggest that SIRF is a robust sensitive method for detection of both replication and repair protein interactions with normal and transiently stalled replication forks.

### Spatiotemporal resolution and single-cell identity in heterogeneic cell populations

**Figure 6.**
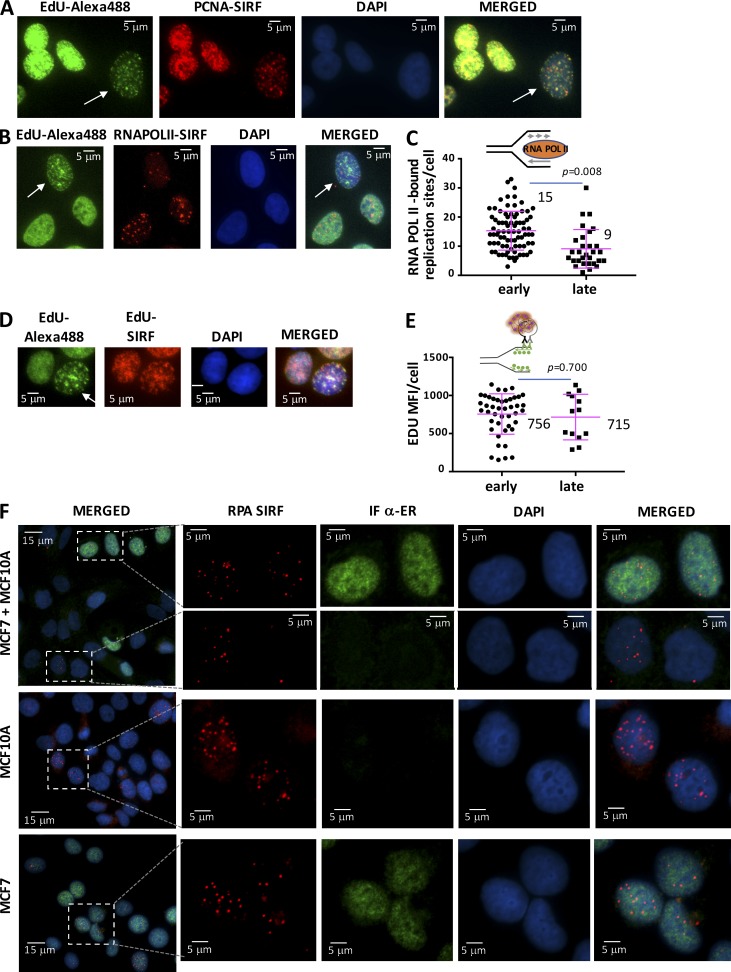
**Multi-parameter SIRF. (A)** Representative images of cells with PCNA-SIRF signals in HAP-1 cells (red), Alexa Fluor 488–EdU staining (green), and DAPI (blue). Alexa Fluor 488–EdU stains allows visualization of late S-phase replication structures (large specks; white arrow). **(B)** Representative images of cells with RNA POL II–SIRF signals in HAP-1 cells (red), Alexa Fluor 488–EdU staining (green), and DAPI (blue). Alexa Fluor 488–EdU stains allows to distinguish between late S-phase replication structures (large specks; white arrow) from early S-phase cells (diffuse green signals). **(C)** Scatter plot of RNA POL II–SIRF signals in early and later S-phase HAP-1 cells as distinguished by Alexa Fluor 488–EdU signal pattern. **(D)** Representative images of cells with EdU-SIRF signals in HAP-1 cells (red), Alexa Fluor 488–EdU staining (green), and DAPI (blue). Alexa Fluor 488–EdU stains allows to distinguish between late S-phase replication structures (large specs, white arrow) from early S-phase cells (diffuse green signals). **(E)** Scatter plot of EdU-SIRF signals in early and later S-phase HAP-1 cells as distinguished by Alexa Fluor 488–EdU signal pattern. **(F)** Representative images of cells with RPA-SIRF signals (red), estrogen-receptor antibody-staining (green), and DAPI (blue) in MCF7 cells (bottom), MCF10A cells (middle), and MCF7+MCF10A co-cultures (top). Bars represent the mean and SD of combined data from repeated experiments. The significance for EdU-SIRF values is derived from the Mann-Whitney statistical test, and the significance for protein-SIRF values is derived from the Mann-Whitney statistical test after normalization to the corresponding EdU-SIRF.

SIRF is an in situ technology with single-cell resolution. In principle, this allows for recording distinct cellular characteristics in addition to the protein–DNA SIRF signals including, among others, cell morphology, cell identity, temporal cell cycle state, and spatial distribution of SIRF signals. Cells in late S-phase can be distinguished from cells in early S-phase by IF. Specifically, cells in late S-phase exhibit large replication foci when labeled with BrdU or Alexa Fluor 488–EdU, whereas these replication markers show diffused signals throughout the nucleus during early S-phase cells (Fox et al., 1991; Watanabe and Maekawa, 2010). We co-clicked EdU with Alexa Fluor 488–azide to mark replication foci, while concomitantly performing a SIRF against replisome factor PCNA (Fig. 6 A). We found that PCNA-SIRF signals overlap with Alexa Fluor 488–EdU signals, and PCNA-SIRF signals greatly followed the spatial pattern seen for early and late S-phase cells. However, of note, the PCNA-SIRF signals resulted in more distinct foci whether cells were in early or late S-phase, with the latter being much more apparent and hence distinguishable with the Alexa Fluor 488–EdU label. Thus, costains, such as Alexa Fluor 488–EdU, can be used to acquire additional information about spatial localization of SIRF signals.

We further validated this approach and tested RNA POL II–SIRF with Alexa Fluor 488–EdU costain to distinguish early from late S-phase cells (Fig. 6 B). Early S-phase cells are associated with greater transcription activity compared with late S-phase cells (Gilbert, 2002; Watanabe and Maekawa, 2010). Consistently, we find significantly more POL II–SIRF signals associated with early S-phase cells compared with late S-phase cells after normalization to EdU-SIRF (Fig. 6, B–E), suggesting RNA POL II is less associated with previously replicated DNA later in S-phase.

Aside from spatiotemporal information, we sought to distinguish individual cell identities in heterogeneic cell populations, which can be another valuable SIRF assay parameter. As proof of principle, we performed RPA-SIRFs in cultures of mixed mammary cells (Fig. 6 F). All MCF7 cells express estrogen receptor (ER) and are readily identifiable by IF with an antibody against ER (Fig. 6 F, bottom). In contrast, MCF10A mammary cells are ER-negative and show no signal with staining against ER (Fig. 6 F, middle). Thus, the ER stain can be used as a biomarker for MCF7 cells when co-culturing MCF7 and MCF10A mammary cells, and RPA-SIRF signals stemming from MCF7 cells can be distinguished from those produced in MCF10A cells (Fig. 6 F, top). Collectively, the data demonstrate that SIRF allows for detecting spatial and temporal cell information as well as the cell identity in heterogeneic cell populations, enabling a multiparameter assay system for fine-tuned phenotypic characterization.

### SIRF for quantitative repair protein detection to stalled forks

**Figure 7.**
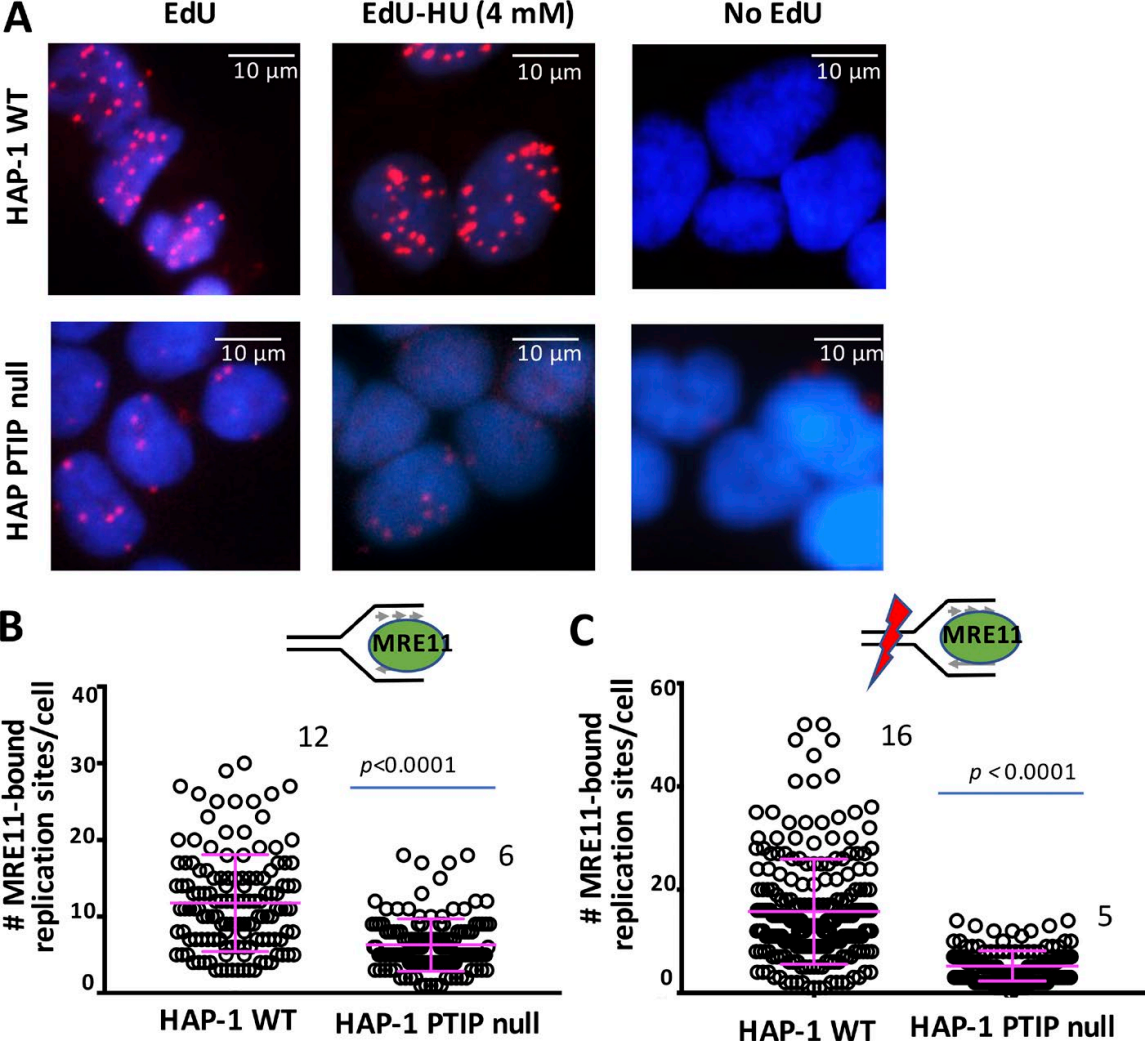
**Reduced MRE11-SIRF in PTIP-defective cells. (A)** Representative images of cells with MRE11-SIRF signals in HAP-1 WT cells (top) and PTIP knockout HAP-1 cells (bottom, PTIP null) treated with EdU, EdU followed by HU (4 mM, 4 h), or no EdU. **(B)** Scatter plot of MRE11-SIRF signals in unperturbed HAP-1 cells and PTIP knockout HAP-1 cells. **(C)** Scatter plot of MRE11-SIRF signals in HAP-1 cells and and PTIP knockout HAP-1 cells treated with EdU followed by HU. Bars represent the mean and SD of combined data from repeated experiments. The significance values are derived from Mann-Whitney statistical analysis after normalization to the corresponding EdU-SIRF.

iPOND technology is a powerful method to detect proteins at stalled replication forks, although it is labor-intensive and requires advanced technical skill and abundant amounts of sample. Using iPOND, data showed that PTIP-deficient mouse embryonic fibroblast (MEF) cells are inefficient in recruiting the DNA repair nuclease MRE11 to stalled DNA replication forks (Ray Chaudhuri et al., 2016). We sought to test and extend from this observation and tested MRE11 recruitment to stalled forks in human HAP-1 cells using SIRF (Fig. 7). We find that MRE11 is efficiently recruited to both ongoing forks and forks stalled with HU in HAP-1 cells (Fig. 7, A–C; and Fig. S3, A and B). In contrast, PTIP knockout HAP-1 cells showed a stark decrease in MRE11 recruitment to both ongoing replication forks and forks that are challenged with HU (Fig. 7, A–C). This loss in signal is not caused by decreased EdU signal, as we find EdU-SIRF signals to be increased in PTIP knockout cells compared with WT HAP-1 cells in either condition (Fig. S3, A and B). Interestingly and supporting previous data (Ray Chaudhuri et al., 2016), MRE11-SIRF in unchallenged primary MEF cells results in scarce MRE11-SIRF signals, whereas MRE11 is readily detected with replication stalling (Fig. S3, C and D), suggesting active MRE11 recruitment to stalled forks. Collectively, the data indicate that SIRF can principally recapitulate data obtained with the iPOND methodology.

### 53BP1 defects increase mutagenic RAD52 and POLθ at stalled forks

**Figure 8.**
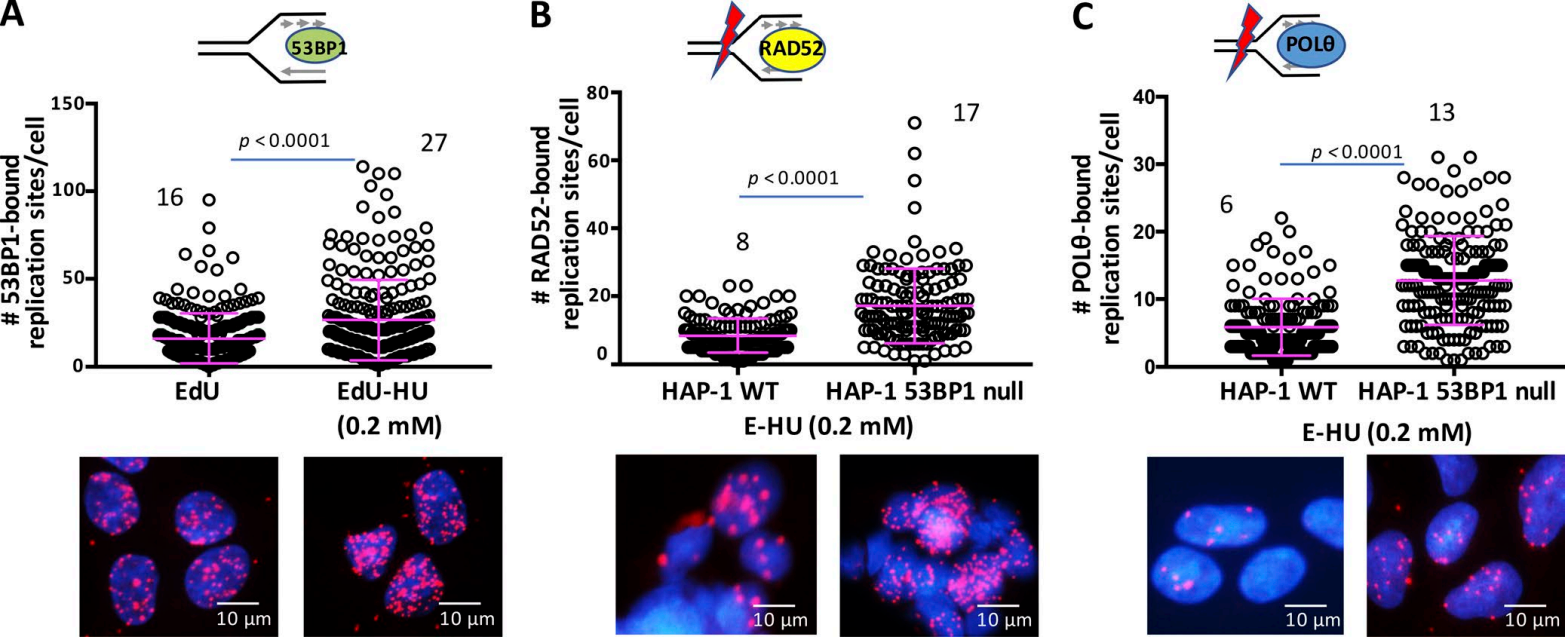
**Increased RAD52- and POLθ-SIRF in 53P1-defective cells. (A)** Scatter plot of 53BP1-SIRF signals in HAP-1 cells treated with EdU or EdU followed by HU, as indicated. **(B)** Scatter plot of RAD52-SIRF signals in HAP-1 and 53BP1 knockout HAP-1 cells (HAP-1 53BP1 null), treated with EdU followed by HU (4 h). **(C)** Scatter plot of POLθ-SIRF signals in HAP-1 and 53BP1 knockout HAP-1 cells (HAP-1 53BP1 null), treated with EdU followed by HU (4 h). Bars represent the mean and SD of combined data from repeated experiments. The significance values are derived from Mann-Whitney statistical analysis after normalization to the corresponding EdU-SIRF.

Our tests of the SIRF assay supported this assay system as a sensitive, quantitative, and efficient method to bring new insight into our current understanding of DNA replication and DNA repair pathway player recruitment to ongoing and stalled replication forks. We thus sought to apply SIRF to better understand DNA repair pathway player hierarchies at replication forks. The p53 binding protein 1 (53BP1) promotes end joining during variable diversity joining and class-switch recombination of immunoglobulins (Ward et al., 2004; Difilippantonio et al., 2008) and is best understood for its DSB repair function during the G1 cell cycle phase. Select studies, however, show 53BP1 associations with DNA replication reactions (Sengupta et al., 2004; Harrigan et al., 2011). We sought to test these observations and performed SIRF assay against 53BP1 in HAP-1 cells and found that 53BP1 is associated with nascent DNA and is increasingly recruited to transiently stalled replication forks (Fig. 8 A and Fig. S4 A).

At DNA breaks, deletion of 53BP1 results in uninhibited resection (Zimmermann and de Lange, 2014), which allows increased RAD51 assembly to DNA and promotes error-free homology-directed repair (HDR; Bouwman et al., 2010; Bunting et al., 2010). We therefore performed SIRF assays against RAD51 and, consistent with the functions of 53BP1 at breaks, we found a significant increase in RAD51 associations to stalled replication forks in 53BP1 knockout HAP-1 cells compared with WT HAP-1 cells (Fig. S4 B) after normalization to EdU-SIRF signals (Fig. S4 C). These data suggest conserved pathway handoffs at transiently stalled forks and DNA breaks with respect to RAD51.

53BP1 antagonizes resection that produces single-stranded DNA overhangs that in principle are substrates for diverse DNA repair pathways including HDR, and mutagenic single-strand annealing or microhomology-mediated end joining (MMEJ; Branzei and Foiani, 2008; Moynahan and Jasin, 2010; Black et al., 2016; Wood and Doublié, 2016). We therefore tested SIRF assays against RAD52. We found starkly increased RAD52 recruitment to transiently stalled forks in 53BP1 knockout HAP-1 cells compared with WT HAP-1 cells (Fig. 8 B and Fig. S4 D; 8 RAD52-bound replication sites per cell in HAP-1 cells and 17 RAD52-bound replication sites per cell in 53BP1 knockout HAP-1 cells). Similarly, POLθ associations were significantly increased in 53BP1-defective HAP-1 cells compared with WT HAP-1 cells after normalization to EdU-SIRF signals (Fig. 8 C and Fig. S4 E; 6 POLθ-bound replication sites per cell in HAP-1 cells and 13 POLθ-bound replication sites per cell in 53BP1 knockout HAP-1 cells). Collectively, we find mutagenic RAD52/single-strand annealing and POLθ/MMEJ pathway recruitment increased in addition to RAD51/HDR at transiently stalled replication forks in the absence of functional 53BP1 by the sensitive SIRF methodology.

## Discussion

Here we describe an in situ methodology for studies of protein association with active and stalled replication forks (SIRF). In SIRF, we combine PLA with click chemistry of nascent DNA replication forks that are labeled with EdU for direct protein–DNA fork interaction. We here show that SIRF is an objective quantitative method with high sensitivity suitable to investigate protein interactions at active, ongoing, and stalled DNA replication forks. Because of its sensitivity and quantitation ability in single cells, SIRF provides a broadly enabling technology that can provide critical new insight into DNA replication and repair processes in cells.

There are key advantages of SIRF over other technologies for protein–DNA fork interactions. We find that SIRF is an in situ procedure with single-cell resolution that allows for fine-scale multiparameter measurements of protein-nascent DNA association detection in heterogeneous cell populations. We further find that DNA repair and replication protein associations with stalled and active replication forks can readily be measured in an unambiguous quantitative fashion. The procedure is performed directly on coverslips or microscope slides and so requires very little starting material (∼10,000 cells per condition). The method follows in principle standard IF procedures; thus, SIRF does not necessitate specialized equipment, but common molecular laboratory equipment and practices suffice. Collectively, we find that SIRF is a sensitive, quantitative, and efficient method that allows the detection of direct protein interactions with nascent DNA at a single-cell resolution.

The single-cell resolution additionally can be expanded with concomitant IF staining, which allows for an added parameter detection. We show that SIRF is suitable to provide additional spatial, temporal, and cell identity information in heterogeneic cell cultures. However, in principle, any parameter that is detectable by microscopy, including but not limited to epigenetic chromatin status or cell morphology, may be combined for multiparameter SIRF. Moreover, because all cells are retained, simultaneous analysis of cells that are not in S-phase may also be interrogated by IF. We anticipate that SIRF will be further developed to include these and other expanded information. DNA replication and repair reactions are at the heart of many diverse biological reactions; thus, SIRF can provide broad applicability for diverse studies of protein associations to nascent DNA.

So far the only method that allows the direct study of protein interactions with active replication forks is the iPOND technology (Sirbu et al., 2011, 2012). This technique remains valuable and has made key advances in the fields of DNA replication and repair by pushing our understanding of replication reactions; however, it is laborious, requires abundant amounts of starting material (100,000,000 cells per condition), and can vary in data quality among cell lines from our laboratory experience with challenging quantitation and limited sensitivity. SIRF at its core is an IF technology in intact cells, and so not all epitopes at the forks may be accessible to antibodies. As such, iPOND by Western blot technology, which utilizes antibodies against denatured proteins, in principle may have an advantage in select instances, albeit we have not encountered this so far. In both instances, the technique relies in part on the relative affinity and specificity of an application-specific antibody. SILAC is an advanced iPOND procedure that replaces Western-blot analysis with mass spectrometry (Sirbu et al., 2013; Cortez, 2017), resulting in a highly quantitative and an unbiased approach. iPOND with SILAC is a discovery approach, which contrasts SIRF and iPOND by Western blot, which are candidate approaches. However, mass spectrometry and the necessary SILAC materials are costly and not necessarily readily accessible. Moreover, by design, iPOND and SILAC detect protein–DNA association changes that are mean changes over an entire cell population. SIRF complements these methods by overcoming some of the current shortcomings; it reduces the required starting material by 10,000-fold, it increases sensitivity because of PLA technology, and provides directly quantitative measurements. Importantly, SIRF allows for analysis and direct visualization of protein-replication fork changes with single-cell resolution among a heterogeneous cell population. As tumors are composed of cells from diverse origins varying in disease contributions and outcome expectation, we expect that SIRF may be in particular useful for examining cancer cell replication reactions and cellular responses in heterogeneous cell populations in tissue and eventually in vivo.

We here show that the obtained SIRF signal is dependent on the proximity of the EdU molecules incorporated into the nascent DNA (Fig. 2). Additionally, our data imply that SIRF controls against EdU alone should be considered for accurate data interpretation when comparing relative SIRF signal changes among cell lines or conditions. We normalize protein-SIRF data to EdU-SIRF data performed in parallel for calculations of statistical significance. In principle, EdU can be clicked to both biotin-azide and Alexa Fluor 488–azide simultaneously, so that Alexa Fluor 488 could also be directly used for EdU normalization (Fig. 6; Sidorova, J., personal communication). However, we find that residually incorporated EdU after washout, such as during replication stalling or chase conditions, results in lesser EdU-SIRF signals compared with Alexa Fluor 488 signals because of a greater distance between EdUs during incorporation of residual EdU after washout and so loss of EdU-SIRF signals. Additionally, co-click of Alexa Fluor 488–azide with biotin-azide can reduce the number of biotins available for protein-SIRF, and further bias the results in select instances (unpublished data; Sidorova, J., personal communication). We thus prefer normalization to EdU-SIRF conditions, albeit normalization should be evaluated based on the experimental setup.

Because nucleotide incorporation rates can differ among cell lines, EdU concentrations should be considered when working with diverse cells. Although both 25 and 125 µM EdU resulted in a robust SIRF signal in HAP-1 cells, 125 µM EdU resulted in significantly more SIRF signals compared with 25 µM when testing either RAD52 or RPA (Fig. S1 C and Fig. S2 A). Our data suggest that higher EdU concentrations reduce the distance between EdU incorporations (Fig. 2). Because PLA interactions are limited to distances no greater than ∼40 nm, our data reflect that high EdU concentrations allow for more effective detection of protein associations with nascent DNA ends, such as seen with RPA and RAD52. Similarly, shorter 4-min EdU pulses, which likely do not change the number of active forks compared with our 8-min EdU pulses, result in virtually identical SIRF data, consistent with detection of fork-end associations (unpublished data). We therefore proceeded with high concentrations of 125 µM EdU to obtain high sensitivity in DNA-end interactions and additionally provide a buffer for the potential difference in cell proliferation rates of various cell types. We were thereby successful in applying the SIRF method in diverse cell types ranging from human cancer cells (HAP-1 and MDA-MB-231; Figs. 2, 3, 4, 5, 6, 7; Fig. S2; and not depicted), to spontaneously immortalized mammary breast cells (MCF10A; Fig. S2), and to primary MEFs (Fig. S3). Nevertheless, we recommend that each cell system should be evaluated with regard to EdU concentrations.

In all our tests, we found that SIRF is sufficiently sensitive to detect proteins that work with the replisome including RPA at ongoing replication forks. This is unlike conventional IF (Fig. 3). In support of our findings are previous studies of the repair protein RAD51, which cannot be readily detected at stalled replication forks by IF, whereas more sensitive techniques involving halogenated DNA pulldown and Western-blot analysis revealed RAD51 association with nascent DNA forks (Petermann et al., 2010a). Interestingly, we find fewer RPA associations with transiently stalled replication forks (low doses of HU) compared with ongoing replication forks. This is not a result of less EdU incorporation as EdU-SIRF signals are increased in transiently stalled cells (Fig. S1 D). This finding is against current common models of continuous helicase unwinding that creates extended single-stranded regions for RPA binding at stalled replication forks based on predominantly in vitro studies with *Xenopus laevis* models (Zeman and Cimprich, 2014). Following this model, we expected increased rather than decreased RPA signals. Although fork uncoupling remains prominent in human cells (Schlacher et al., 2011; Zellweger et al., 2015), single-stranded DNA generation is more limited compared with *Xenopus* systems (Zellweger et al., 2015). Additionally, ∼30% of forks reverse, which in turn may limit extensive exposure of single-stranded DNA (Zellweger et al., 2015). Last, low HU concentrations may permit limited restart and therefore chasing of the RPA away from the nascent labeled DNA. Thus, it will be exciting to use SIRF assays to gain more insight into replication reactions and protein dynamics at transient and distinct replication structures in cells.

Although also increased in SIRF assays, the difference in RPA signals between high HU and no HU reactions is greater with IF. We confirmed that EdU concentrations are within the linear range (Fig. S1), which otherwise would result in saturated SIRF signals and could have explained the seeming difference in results. Alternatively, IF may detect signal only above a threshold of fluorescence intensity, which could be overcome by signal amplification with PLA. As RPA binds to the parental strands of the resected DNA strands, RPA-SIRF most probably only detects RPA interactions in proximity to the tips of nascent DNA, but will be blind to protein associations with unlabeled strands, which should be considered during the experimental design. Collectively, although complementary, both current and new technologies including IF, iPOND, and SIRF, have advantages and disadvantages within distinct scientific interrogations.

SIRF successfully recapitulates previous findings of impaired MRE11 nuclease recruitment to stalled forks in PTIP-defective cells (Ray Chaudhuri et al., 2016; Fig. 7). In addition to stalled forks, SIRF showed a weak MRE11 recruitment to both unchallenged and stalled replication forks in HAP-1 PTIP knockout cells compared with WT HAP-1 cells (Fig. 7). In contrast, and consistent with previous studies, we were unable to detect robust MRE11-SIRF signals in unchallenged primary MEF cultures, whereas MRE11 is readily recruited to stalled DNA forks in these cells (Fig. S3). Thus, MRE11 recruitment in HAP-1 cells without external replication stress feasibly could be a reflection of increased intrinsic replication stress in cancer cells compared with primary cells, or alternatively differences between species.

We applied SIRF to test associations of the DNA repair protein 53BP1 to ongoing and stalled replication forks. Although 53BP1 is best understood for its DSB repair function during G1 of the cell cycle and for telomere protection (Difilippantonio et al., 2008; Zimmermann and de Lange, 2014), it has been reported that upon replication stalling, 53BP1 colocalizes with BLM (Bloom syndrome helicase; Sengupta et al., 2004), a protein that is required for efficient replication restart (Davies et al., 2007; Schlacher et al., 2011). Moreover, DNA damage sites that remain unresolved during S-phase are marked by 53BP1, which form “53BP1 bodies” that remain throughout one cell cycle until they are resolved during the following S-phase (Lukas et al., 2011). We here show that 53BP1 indeed directly associates with replication forks (Fig. 8 A). This result opens the door for new investigations of 53BP1, and perhaps other canonical nonhomologous end-joining (NHEJ) factors, in maintaining replication fork reactions. During DSB repair, 53BP1 plays a pivotal role in pathway choice; 53BP1 inhibits DNA end resection necessary for HDR and so promotes NHEJ at DNA breaks (Bouwman et al., 2010; Bunting et al., 2010). This property has been suggested to have important implications for acquisition of chemoresistance; the breast cancer tumor suppressor BRCA1 promotes HDR by mediating DNA end resection and RAD51 loading. BRCA1 defective cells can’t efficiently repair DSBs by HDR and so are sensitive to break-inducing agents during S-phase, including the chemotherapeutic agent cisplatin. Simultaneous deletion of 53BP1 eliminates the DNA end resection antagonist and restores RAD51 loading and HDR despite BRCA1 defects. 53BP1 deletion or mutations thus allow repair and therefore resistance in BRCA1 defective tumor cells, which is a proposed prominent resistance pathway in breast cancers (Bouwman et al., 2010; Bunting et al., 2010). As seen for breaks, we furthermore here find that RAD51 associations are increased at stalled forks in 53BP1 knockout HAP-1 cells. Unexpectedly, we find a stark increase in both RAD52 and POLθ recruitment to stalled replication forks in the absence of 53BP1. These findings support and extend our understanding of DNA repair pathway hierarchies at DNA breaks, where inactivation of classical NHEJ results in both increased HDR and in RAD52-dependent single-strand annealing (Stark et al., 2004). Moreover, a recent study finds RAD52 damage foci in response to radiation damage increased in 53BP1/BRCA1-defective cells, contributing to enhanced survival after radiation damage (Ochs et al., 2016). The simultaneous up-regulation of mutagenic and error prone pathways at stalled DNA replication forks suggests that in addition to restoration of BRCA-related HDR functions, 53BP1 mutations and deletions may result in increased mutagenic pathways that in principle could drive tumor mutagenesis and progression. As most chemotherapeutics target DNA replication forks, it will be of great interest to further dissect 53BP1 roles in antagonizing POLθ/MMEJ at replication forks and examine how such roles impact chemotherapy sensitivity and resistance.

In sum, we describe here a SIRF procedure for single-cell–level analysis of protein association with nascent DNA replication forks. SIRF is a sensitive, quantitative, efficient, accessible, and comparatively inexpensive method with single-cell resolution that requires little experimental starting material. Although SIRF lends itself for studies of DNA replication dynamics and DNA repair-protein reactions at replication forks, its application is not limited to such areas. Indeed, a similar technique has been reported for the detection of chromatin and transcription factors associated to previously replicated DNA (Petruk et al., 2012, 2017). Importantly, it may be adapted for diverse applications including cell developmental, epigenetic, in vivo cancer cell progression, and therapy responses with the key added benefit of allowing objective and quantitative multiparameter measurements within heterogeneous cell populations.

## Materials and methods

### Reagents, cell lines, and culture conditions

EdU, HU, thymidine, camptothecin, copper sulfate, ascorbic acid, Duolink PLA Probes (anti–mouse plus and anti–rabbit minus), Duolink detection reagent red, goat serum, and cOmplete Mini EDTA-free protease inhibitor cocktail were obtained from Sigma-Aldrich. Biotin azide (PEG4 carboxamide-6-azidohexanyl biotin), Alexa Fluor 488 azide, Texas red–conjugated neutravidin, and DAPI were obtained from Life Technologies. Paraformaldehyde was obtained from Electron Microscopy Sciences. Nunc Lab-Tek chamber slides and Prolong Gold antifade mountant were obtained from Thermos Fisher Scientific (Life Technologies).

Antibodies used for DNA fibers, IF, and SIRF are as follows: goat biotinylated anti-avidin antibody (Vector Laboratories), mouse anti-biotin (BN-34, 1:100; Sigma-Aldrich), rabbit anti-biotin (D5A7, 1:200; Cell Signaling), rabbit anti-RPA70 (EPR3472, 1:500; Abcam), mouse anti-PCNA (PC10, 1:100; Santa Cruz Biotechnology), rabbit anti-CAF1 (EPR5576, 1:200; Abcam), mouse anti-MRE11 (12D7, 1:200; Abcam), mouse anti-RAD52 (F7, 1:100; Santa Cruz Biotechnology), rabbit anti-POLθ (1:100; Abcam), mouse anti-RAD51 (14B4, 1:200; Abcam), mouse anti-ER (F10, 1:100; Santa Cruz Biotechnology), and rabbit anti–RNA POL II (1:500; Abcam).

HAP-1 parental, HAP-1 TP53BP1 knockout, and HAP-1 PTIP knockout cells (Horizon Discovery) were grown in Iscove’s modified Dulbecco’s medium (Life Technologies) supplemented with 10% fetal bovine serum (Gemini Bio-Products) and 100 U/ml Pen-Strep (Life Technologies). MCF10A cells were grown in DMEM:F12 (Life Technologies) supplemented with 5% horse serum (Life Technologies), 20 ng/ml EGF (Peprotech), 0.5 µg/ml hydrocortisone (Sigma-Aldrich), 100 ng/ml cholera toxin (Sigma-Aldrich), 10 µg/ml insulin (Sigma-Aldrich), and 100 U/ml Pen-Strep (Life Technologies). Primary MEFs were obtained from the Guillermina Lozano laboratory (University of Texas MD Anderson Cancer Center, Houston, TX) and were generated from C57BL/6J mice with mixed sex background. MEFs were grown in DMEM (Life Technologies) supplemented with 10% fetal bovine serum (Gemini Bio-Products), 100 U/ml Pen-Strep, and 2 mM glutamine (Life Technologies). MDA-MB-231 breast cancer cells were grown in DMEM:F12 (Life Technologies), 10% fetal bovine serum (Gemini Bio-Products), and 1.5% Hepes. Cell lines have been authenticated by short tandem repeat profile analysis and genotyping. All cells were grown at 37°C and 5% CO_2_.

### SIRF assay

10,000 cells grown in log-phase were plated the day before the experiment at 50–60% confluence onto microscope chamber slides. On the day of the experiment, the wells were checked for appropriate confluency ensuring log-phase growth of the cells. Cells were incubated with 125 µM EdU for 8 min unless indicated otherwise in the figures and legends, and fixed with 2% PFA in PBS (pH 7.4) for 15 min at room temperature (PFA should be handled with caution inside a chemical cabinet). For conditions with replication stalling and thymidine chases, EdU was removed and slides were washed two times with PBS before addition of media with HU (0.2 or 4 mM) or thymidine (100 µM; see figure legends for duration) before fixation (media needs to be preequilibrated to 37°C before treatments, and handling of treatments should be performed quickly to avoid extended times outside the cell incubator, which disrupts cell proliferation). After fixation, PFA was discarded, chambers were removed from slides, and slides were washed in Coplin jars filled with PBS two times for 5 min each (it is important to wash with ample amounts of PBS to not adversely affect downstream processing). Cells were next permeabilized by placing slides in Coplin jars containing 0.25% Triton X-100 in PBS for 15 min at room temperature. Slides were washed in PBS twice for 5 min each. Click reaction cocktail was freshly prepared as follows: 2 mM copper sulfate, 10 µM biotin-azide, and 100 mM sodium ascorbate were added to PBS in that order and mixed well (1 M sodium ascorbate solution is made fresh every time before preparing the click reaction cocktail). Slides were placed in a humid chamber, and click reaction cocktail was added to the slides (30 µl/well) and incubated at room temperature for one hour. Alternatively, biotin-azide and Alexa Fluor 488–azide (1:10, total of 10 µM) may be added to the click reaction (the humid chamber was prepared by lining a slide box with moist Kim wipes; slides were laid flat in the slide box facing up; after addition of click reaction cocktail, plastic coverslips were placed onto the slides during incubation, making sure to avoid air bubbles). After the click reaction, slides were washed in a Coplin jar containing PBS for 5 min. Slides were placed back in the humid chamber and blocked with blocking buffer (10% goat serum and 0.1% Triton X-100 in PBS) for 1 h at room temperature. Primary antibodies were diluted in blocking buffer, dispensed onto slides (30–40 µl/well), and incubated at 4°C overnight in a humid chamber. (Excess blocking buffer was flicked off before addition of primary antibody solution. Either mouse anti-biotin or rabbit anti-biotin antibody was used in conjunction with the respective antibody for the protein of interest. For SIRF costaining with IF, primary and secondary antibody incubation was performed as outlined below for IF before PLA.) Slides were washed three times with wash buffer A (0.01 M Tris, 0.15 M NaCl, and 0.05% Tween 20, pH 7.4) for 5 min each. Duolink In Situ PLA probes anti–mouse plus and anti–rabbit minus were diluted 1:5 in blocking solution (10% goat serum and 0.1% Triton X-100 in PBS), dispensed onto slides (30 µl/well), and incubated for 1 h at 37°C. Slides were again washed in 60 ml wash buffer A solution three times for 5 min each. Ligation mix was prepared by diluting Duolink ligation stock (1:5) and ligase (1:40) in high-purity water. (Keep ligase in a freezing block at −20°C and vortex ligation stock before use, making sure to dissolve any precipitate). Slides were placed back in the humid chamber, and ligation mix was dispensed onto slides (30 µl/well) and incubated at 37°C for 30 min. Slides were washed in 60 ml wash buffer A two times for 2 min each. Amplification mix was prepared by diluting Duolink amplification stock (1:5) and rolling circle polymerase (1:80) in high-purity water. (Enzyme was kept in a freezing block at −20°C and exposure of amplification stock to light was limited.) Slides were placed back in the humid chamber, and amplification mix was dispensed onto slides (30 µl/well) and incubated at 37°C for 100 min (it is critical to keep the incubation at 100 min). Slides were washed in 60 ml wash buffer B solution (0.2 M Tris and 0.1 M NaCl) three times for 10 min each and one time in 0.01× diluted wash buffer B solution for 1 min. Slides were placed back in humid chamber, and DAPI solution was dispensed onto slides (30 µl/well, 1 µg/ml; Life Technologies) and incubated at room temperature for 5 min. Slides were washed in 60 ml PBS for 5 min. Excess liquid was tapped off the slides, and one drop of Prolong Gold antifade (Life Technologies) was added to each well to mount slides with glass coverslips (1.5 mm; Thermo Fisher Scientific; avoid air bubbles and gently tap to eliminate any remaining air bubbles). The slides were kept in the dark overnight to cure. Slides were imaged using a Nikon Eclipse Ti-U inverted microscope and analyzed using Nikon NIS elements software and Duolink quantification tool software (Sigma-Aldrich).

### EdU fiber analysis

100,000 cells were plated the day before the experiment. The day of the experiment, the wells were checked for appropriate confluency ensuring log-phase growth of the cells. Cells were incubated with EdU at indicated concentrations for 25 min, harvested, and resuspended in PBS. Cell suspensions were lysed on a microscope slide in 1:6 lysis buffer (20 mM Tris-Cl, 50 mM SDS, and 100 mM EDTA). Cells were allowed to lyse for 5.5 min before spreading DNA by gravity. Slides were fixed in methanol/acetic acid (3:1) for 3 min and air-dried. EdU fibers were biotinylated using click chemistry with biotin-azide as described above. Slides were blocked with 10% goat serum and 0.1% Triton X-100 in PBS, and biotinylated EdU was subsequently marked by immunostaining using Texas red–conjugated neutravidin followed by a biotinylated anti-avidin antibody, and staining was repeated twice. For PLA fibers, slides were blocked in 10% goat serum/0.1% Triton X-100 after biotinylation and incubated with a mouse anti-biotin antibody (Sigma-Aldrich) and rabbit anti-biotin antibody (Cell Signaling) overnight at 4°C. Duolink PLA (Life Technologies) was performed on the whole slide as described above. Slides were mounted as above and analyzed using an LSM800 Aisyscan microscope (Zeiss) and ImageJ software.

### Pulsed-field gel electrophoresis (PFGE) analysis

PFGE analysis to determine DSB formation was performed as previously described (Zellweger et al., 2015). In brief, log-phase cells were exposed to genotoxic stresses as indicated in Fig. S1 B. 1,000,000 cells each were mixed with 1% low melting point agarose in agarose plugs, digested with digestion mix (0.5 M EDTA, 1% sarcosyl, and 1 mg/ml proteinase K) for 48 h at 50°C with gentle shaking, washed with Tris-EDTA buffer while rotating overnight at 4°C, and resolved using pulse-field electrophoreses. The gel was stained with ethidium bromide and imaged.

### IF

10,000 HAP-1 cells were plated the day before the experiment in microscope chamber slides. The day of the experiment, the wells were checked for appropriate confluency, ensuring log-phase growth of the cells. Cells were treated with HU for 4 h (0.2 or 4 mM) or camptothecin for 1 h (1 µM) as indicated, preextracted on ice using ice-cold CSK buffer (10 mM Pipes, pH 6.8, 300 mM sucrose, 50 mM NaCl, 3 mM EDTA, 0.5% Triton X-100, and 1× complete miniprotease inhibitor) for 5 min and fixed in 2% paraformaldehyde at room temperature for 15 min. Subsequently, cells were permeabilized with 0.25% Triton X-100 in PBS for 15 min and blocked with 10% goat serum and 0.1% Triton X-100 in PBS for 1 h. Primary antibodies against RPA or RAD52 were incubated at room temperature in a humid chamber for 1 h, followed by three PBS washes and subsequent incubation with secondary antibodies (conjugated to Alexa Fluor 546 and 488, respectively) for 1 h at room temperature. Slides were washed in PBS three times and counterstained with DAPI (0.1 µg/ml), rinsed with PBS, and mounted with Prolong Gold antifade mountant. Slides were imaged using an Eclipse Ti-U inverted microscope and analyzed using NIS elements software.

### Imaging and statistical analyses

For SIRF and IF assays, slides were imaged using an Eclipse Ti-U inverted microscope with an Andor Zyla sCMOS camera with a Plan Apochromat objective lens at 40× magnification (0.95 NA, 25°C, in air imaging media). PLA and SIRF signals were counted using either Duolink quantification tool software or NIS elements software. Alternatively, when SIRF signals were too plentiful to be distinguished, the mean MFI/cell was calculated using NIS elements software. Additionally, the MFI/SIRF signal can be used as an additional parameter. 100–300 nuclei were counted for each condition. A minimum of six image fields was acquired for each condition, and the data presented is a compilation of two to four biological replicates. The data were further analyzed using GraphPad Prism version 6 to calculate the mean and SD. For DNA spreads and EdU SIRFs, the significance was calculated using the Mann-Whitney statistical test as indicated in the respective figures and figure legends. For protein SIRFs, the data were normalized to the mean of the corresponding EdU-SIRFs before Mann-Whitney statistical testing. As an alternative statistical test for data with great variance in EdU, a *t* test to determine the *z* score and p-value for significance may be performed using the following equation: *z* = [mean (EdU-SIRF1) − mean (EdU-SIRF2)] − [mean (SIRF1) − mean (SIRF2)]/√[variance (EdU-SIRF1)/*n* + variance (EdU-SIRF2)/*n* + variance (SIRF1)/*n* + variance (SIRF2)/*n*], where *n* is the number of measurements. SIRF data are presented as a scatter dot column plot marking the mean with a horizontal bar and the SD for the error bars. IF data are presented as column bar graph marking the mean and the SEM for the error bars.

DNA fibers were imaged using an LSM800 Aisyscan microscope with an LSM800 Airyscan detector (Zeiss) and analyzed using a Plan Apochromat objective lense at 63× magnification (1.4 NA, 25°C, in oil imaging media) and Zen2 software version 2.3 for Airyscan processing. 100 fibers were scored from three image fields each of two to three biological replicates. The data were further analyzed using ImageJ software for length measurements and GraphPad Prism version 6 to calculate the mean, SD, and significance using the Mann-Whitney statistical test as indicated in the respective figures and figure legends. The data are presented as a scatter dot column plot marking the mean with a horizontal bar and the SD for the error bars.

### Online supplemental material

Fig. S1 shows representative images of RPA-SIRF, PFGE analysis for DSB breaks, RPA-SIRF at varying EdU concentrations, and EdU-SIRF controls for corresponding RPA-SIRF. Fig. S2 shows RAD52-SIRF at varying EdU concentrations, EdU-SIRF controls for corresponding RAD52-SIRFs, and RAD52-SIRF in MDA-MB-231 and MCF10A cells with respective EdU-SIRF controls. Fig. S3 shows EdU-SIRF controls correspond to Fig. 7 (Band C), and MRE11-SIRF and corresponding EdU-SIRF controls in primary MEFs. Fig. S4 shows RAD51-SIRF and corresponding EdU-SIRF controls, and EdU-SIRF controls corresponding to Fig. 8.

## Supplementary Material

Supplemental Materials (PDF)
