# Correction: SIRF: Quantitative in situ analysis of protein interactions at DNA replication forks

**DOI:** 10.1083/JCB.20170912103212018c

**Published:** 2018-04-02

**Authors:** Sunetra Roy, Jessica W. Luzwick, Katharina Schlacher

Vol. 217, No. 4, April 2, 2018. 10.1083/jcb.201709121.

After online publication, the authors were made aware of a previously published technique for the detection of chromatin and transcription factors associated to previously replicated DNA, which is similar to the SIRF technique and which they wish to cite.

A corrected version of the Discussion appears below:

“Although SIRF lends itself for studies of DNA replication dynamics and DNA repair-protein reactions at replication forks, its application is not limited to such areas. Indeed, a similar technique has been reported for the detection of chromatin and transcription factors associated to previously replicated DNA (Petruk et al., 2012, 2017). Importantly, it may be adapted for diverse applications including cell developmental, epigenetic, in vivo cancer cell progression, and therapy responses with the key added benefit of allowing objective and quantitative multiparameter measurements within heterogeneous cell populations.”

Both the HTML and PDF versions of the article have been corrected. This error appears only in PDF versions downloaded on or before March 22, 2018.
