# Supplementary material for: SIRF: Quantitative in situ analysis of protein interactions at DNA replication forks
Source: J Cell Biol. 2018 Apr 2;217(4):1521–36. doi: 10.1083/jcb.201709121 (PMC5881507; doi:10.1083/jcb.201709121)
Supplement: Supplemental Materials (PDF) [file JCB_201709121_sm.pdf]

Supplemental material

Roy et al., https://doi.org/10.1083/jcb.201709121

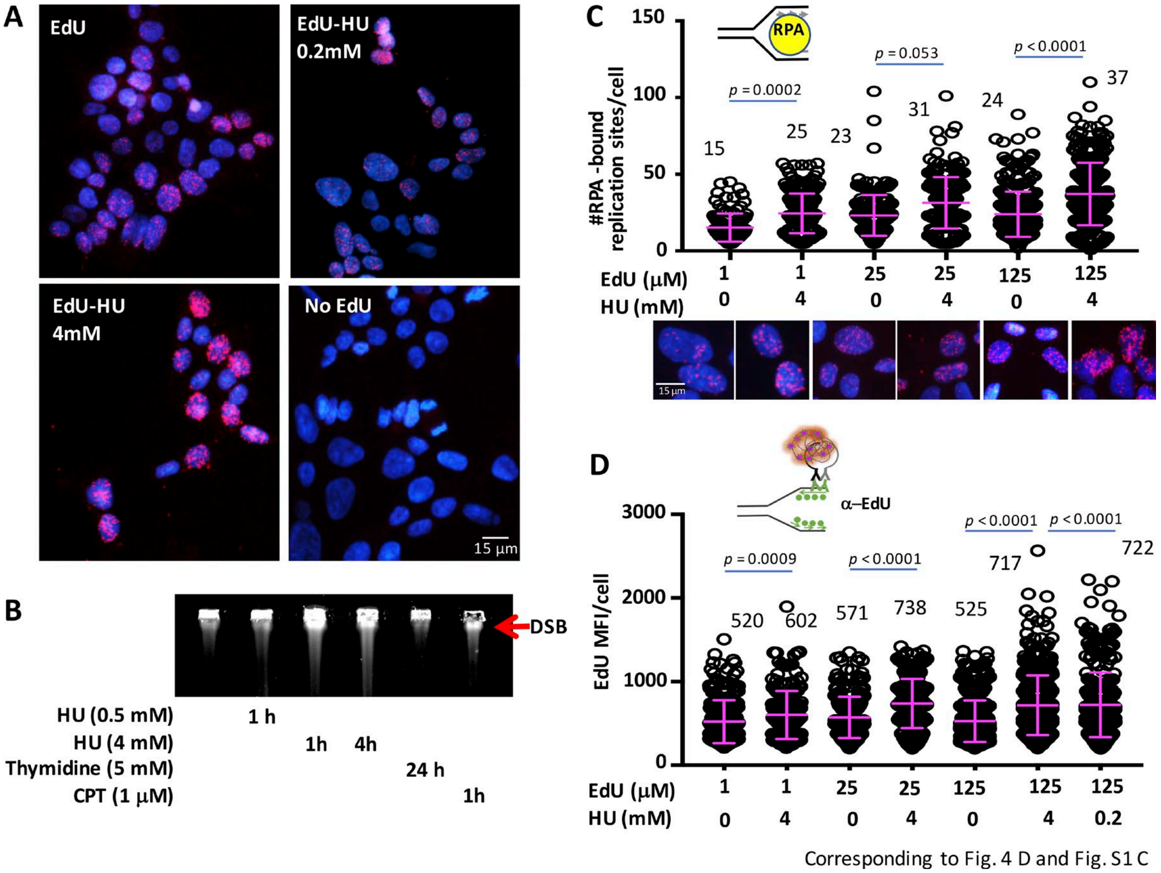

Figure S1. **RPA SIRF at varying EdU concentrations. (A)** Representative images (40× magnification) of RPA-SIRF in HAP-1 cells treated with 125 µM EdU for 8 min followed by HU (0.2 and 4 mM) for 4 h and no EdU as indicated. Cells were immediately fixed and proceeded for SIRF assay according to protocol (see Materials and methods). **(B)** PFGE gel analysis of DSB formation upon genotoxic stresses in U2OS cells. Low concentrations of HU (0.5 mM) and prolonged thymidine treatment result in little to no DSB formation as indicated by limited gel-entry of the DNA. In contrast, prolonged HU exposure of high concentrations of HU (4 mM, 4 h) or camptothecin (1 µM, 1 h) promotes readily detectible DSB formation. Increasing times of high HU concentrations (1 vs. 4 h) promotes increased DSB formation. **(C)** Scatter plot of RPA-SIRF signals in HAP-1 cells, treated with EdU (1, 25, or 125 mM) followed by HU (4 mM, 4 h). Bars represent the mean and SD of combined data from repeated experiments. The significance for RPA-SIRF values is derived from the Mann-Whitney statistical test after normalization to the corresponding EdU-SIRF. Note that scatter plots of 125 mM EdU conditions are replotted from Fig. 4 D for better illustration. **(D)** Scatter plot of EdU-SIRF data points corresponding to C and Fig. 4 D. The significance was derived from the Mann-Whitney statistical test.

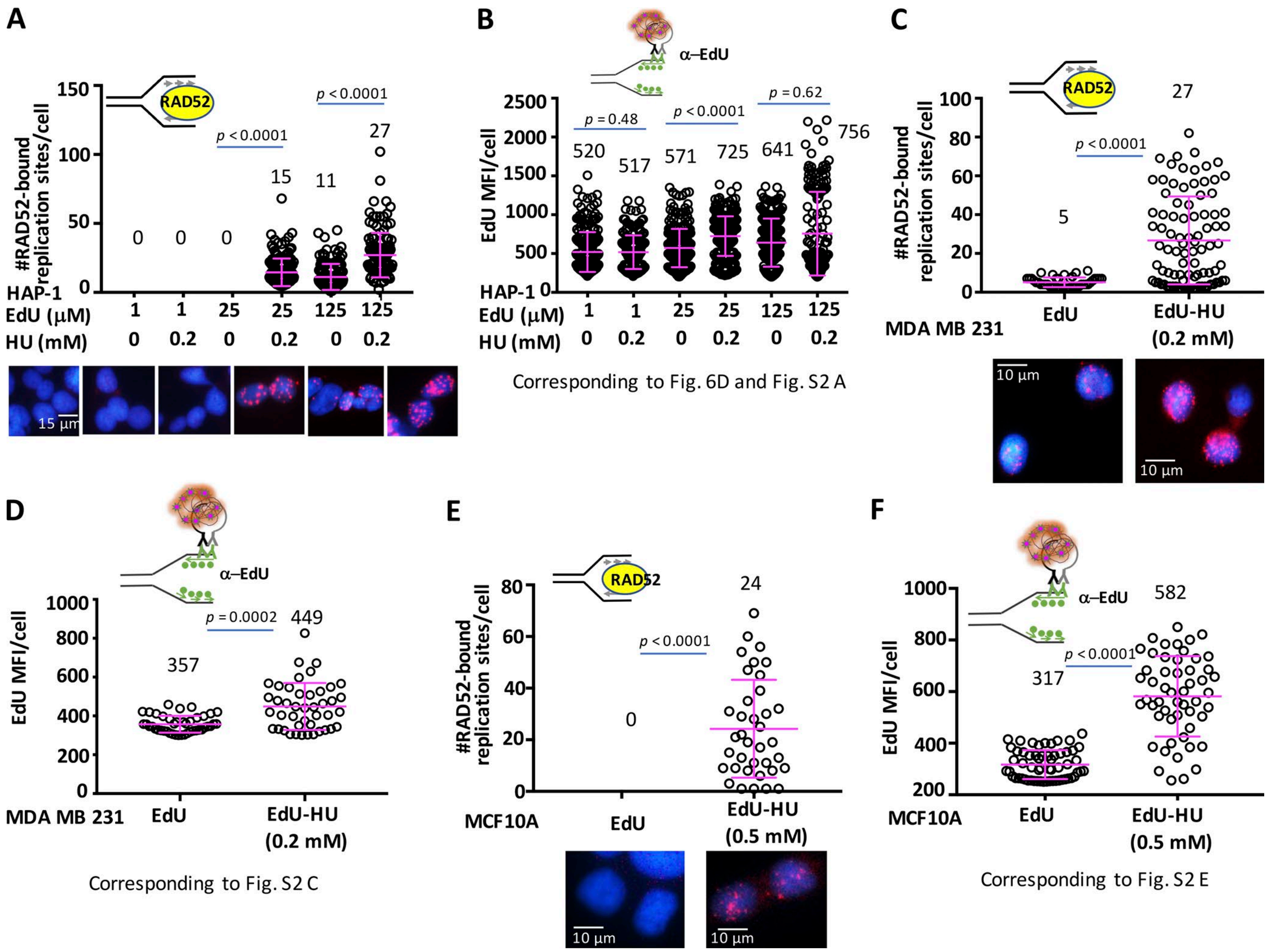

Figure S2. **RAD52 SIRF at varying EdU concentrations and with varying cell types. (A)** Scatter plot of RAD52-SIRF signals in HAP-1 cells, treated with EdU (1, 25, or 125 mM) followed by HU (0.2 mM, 4 h). Bars represent the mean and SD of combined data from repeated experiments. Bottom: Representative images. Note that scatter plots of 125 mM EdU conditions are replotted from Fig. 5 D for better illustration. **(B)** Scatter plot of EdU-SIRF data points corresponding to Fig. 5 D and Fig. S2 A in HAP-1 cells treated with EdU (1, 25, or 125 mM), with and without HU (0.2 mM, 4 h). **(C)** Scatter plot of RAD52-SIRF data points in unperturbed MDA-MB-231 cells and cells treated with EdU followed by HU (0.2 mM, 4 h). Bottom: Representative images. **(D)** Scatter plot of EdU-SIRF data points corresponding to Fig. S2 C in unperturbed MDA-MB-231 cells and cells treated with EdU followed by HU (0.2 mM, 4 h). **(E)** Scatter plot of RAD52-SIRF data points in unperturbed MCF10A cells and cells treated with EdU followed by HU (0.2 mM, 4 h). Bottom: Representative images. **(F)** Scatter plot of EdU-SIRF data points corresponding to Fig. S2 E in unperturbed MCF10A cells and cells treated with EdU followed by HU (0.2 mM). Bars represent the mean and SD of combined data from repeated experiments. The significance for EdU-SIRF values is derived from the Mann-Whitney statistical test, and the significance for protein-SIRF values is derived from the Mann-Whitney statistical test after normalization to the corresponding EdU-SIRF.

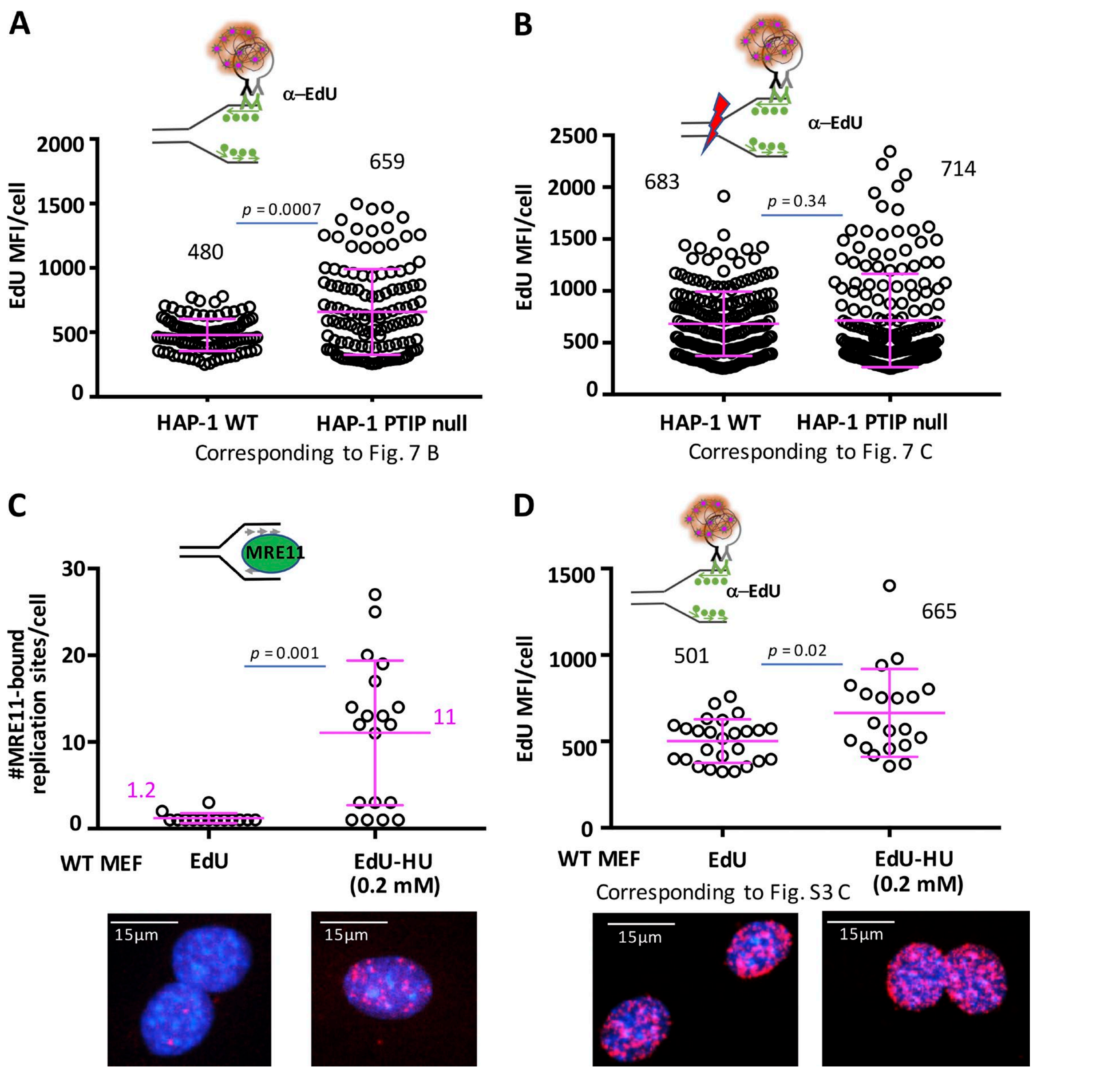

Figure S3. **MRE11 SIRF in primary MEFs. (A)** Scatter plot of EdU-SIRF data points in unperturbed HAP-1 WT and PTIP null HAP-1 cells corresponding to Fig. 7 B. **(B)** Scatter plot of EdU-SIRF data points in HAP-1 WT and PTIP null HAP-1 cells treated with EdU followed by HU (4 mM, 4 h) corresponding to Fig. 7 C. **(C)** Scatter plot of MRE11-SIRF data points in unperturbed primary MEF cells and cells treated with EdU followed by HU (0.2 mM, 4 h). **(D)** Scatter plot of EdU-SIRF data points corresponding to Fig. S3 C in unperturbed primary MEFs and cells treated with EdU followed by HU (0.2 mM, 4 h). Bottom: Representative images. Bars represent the mean and SD of combined data from repeated experiments. The significance for EdU-SIRF values is derived from the Mann-Whitney statistical test, and the significance for protein-SIRF values is derived from the Mann-Whitney statistical test after normalization to the corresponding EdU-SIRF.

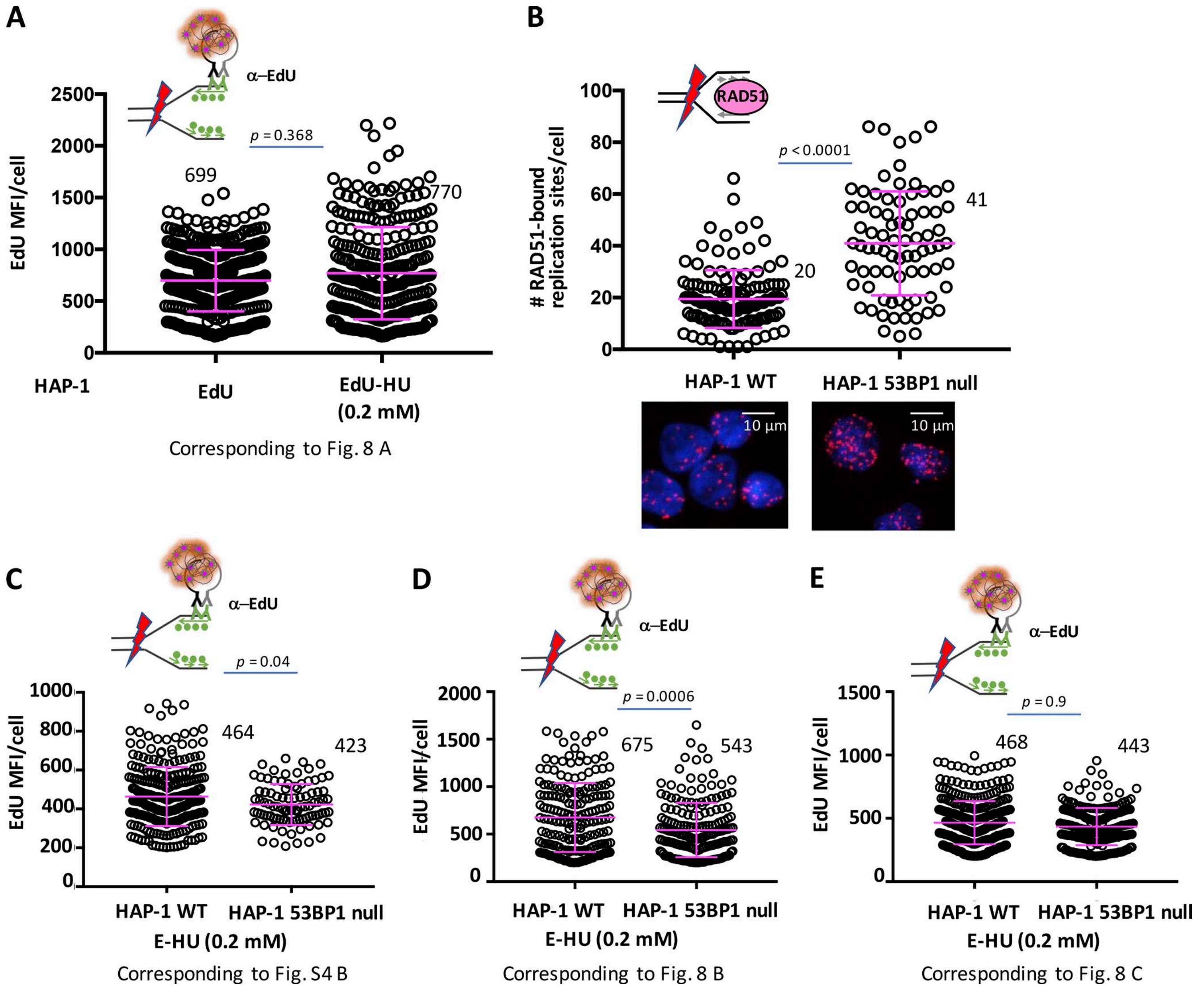

Figure S4. **RAD51 and EdU SIRF in 53BP1 null cells. (A)** Scatter plot of EdU-SIRF data points corresponding to Fig. 8 A of HAP-1 cells treated with EdU or EdU followed by HU (0.2 mM, 4 h). **(B)** Scatter plot of RAD51-SIRF data points of HAP-1 WT and 53BP1 null HAP-1 cells treated with EdU followed by HU (0.2 mM). Bottom: Representative images. **(C)** Scatter plot of EdU-SIRF data points corresponding to Fig. S4 B of HAP-1 and 53BP1 null HAP-1 cells treated with EdU followed by HU (0.2 mM, 4 h). **(D and E)** Scatter plot of EdU-SIRF data points corresponding to Fig. 8 (B and C), respectively, of HAP-1 WT and 53BP1 null HAP-1 cells treated with EdU followed by HU (0.2 mM, 4 h). The significance for EdU-SIRF values is derived from the Mann-Whitney statistical test, and the significance for RAD51-SIRF values is derived from the Mann-Whitney statistical test after normalization to the corresponding EdU-SIRF.
